# Fuzziness and noise in nucleosomal architecture

**DOI:** 10.1093/nar/gku165

**Published:** 2014-02-27

**Authors:** Oscar Flores, Özgen Deniz, Montserrat Soler-López, Modesto Orozco

**Affiliations:** ^1^Institute for Research in Biomedicine (IRB Barcelona), Baldiri Reixac 10-12, 08028 Barcelona, Spain, ^2^Joint IRB-BSC Program in Computational Biology, Baldiri Reixac 10-12, 08028 Barcelona, Spain and ^3^Department of Biochemistry and Molecular Biology. University of Barcelona, 08028 Barcelona, Spain

## Abstract

Nucleosome organization plays a key role in the regulation of gene expression. However, despite the striking advances in the accuracy of nucleosome maps, there are still severe discrepancies on individual nucleosome positioning and how this influences gene regulation. The variability among nucleosome maps, which precludes the fine analysis of nucleosome positioning, might emerge from diverse sources. We have carefully inspected the extrinsic factors that may induce diversity by the comparison of microccocal nuclease (MNase)-Seq derived nucleosome maps generated under distinct conditions. Furthermore, we have also explored the variation originated from intrinsic nucleosome dynamics by generating additional maps derived from cell cycle synchronized and asynchronous yeast cultures. Taken together, our study has enabled us to measure the effect of noise in nucleosome occupancy and positioning and provides insights into the underlying determinants. Furthermore, we present a systematic approach that may guide the standardization of MNase-Seq experiments in order to generate reproducible genome-wide nucleosome patterns.

## INTRODUCTION

Eukaryotic chromatin is organized in a compact, precisely regulated, but yet not fully understood manner. Nucleosome is the fundamental structural unit of this compaction (1,2), formed by the wrapping of 147-bp double-stranded DNA in 1 and ¾ left-handed superhelix around a histone octamer (3). The presence of histone proteins determines the accessibility of DNA to other interacting proteins and plays a role in altering mutation rate (4), in determining exon architecture (5) and in regulation of transcription (6,7).

Genome-wide studies of micrococcal nuclease (MNase) susceptibility have revealed that nucleosomes are not randomly placed across genome but they are rather enriched in certain areas while depleted in others (8–13). The most pervasive regions depleted in nucleosomes appear upstream the transcription start site (TSS), at gene promoters. Such nucleosome-free regions (NFRs) are often flanked by two nucleosomes, one very strongly located downstream TSS (+1 position), and a second one more weakly located upstream (−1 position) (14,15,8,10,13). The integrity of this organization seems to be crucial for a correct gene regulation (16–18), since the introduction of a high-affinity nucleosome binding sequence in a promoter region can inhibit transcription (19).

Despite the tremendous amount of studies, the underlying mechanisms governing *in vivo* nucleosome positioning still remain elusive. Three main models have been postulated as determinants of nucleosome positioning: (i) statistical positioning, suggesting that a barrier favors deposition of a well-positioned nucleosome, typically after a NFRs, which in turn forces the periodic positioning of the neighboring nucleosomes (20,21); (ii) the intrinsic properties of naked DNA that favors histone binding in certain sequences (8,12,22–28) and (iii) DNA-interacting proteins like transcription factors (TFs) (11,29–31), which force nucleosome depletion in certain regions. Notably, there is still controversy among these three models, since all of them seem supported by experimental findings (32–36).

Systematic analyses of nucleosome dynamics still remain a challenge, particularly in determining the exact positioning, fuzziness and affinity (37–42). The struggle partially arises from the paucity of consistent nucleosome maps, even in the same model organisms. Thus, different studies show quite similar average nucleosome profiles, but individual nucleosome positioning might differ remarkably, so that very well-positioned nucleosomes detected in one study might appear as fuzzy or simply absent in another (43–46). As an example, a recent study showed that Pearson’s correlation coefficients range from 0.2 to 0.45 among different *in vivo* nucleosome profile datasets, indicating that in average <10% of nucleosome positioning is actually reproduced by different studies (47). The extreme variability among datasets, not only makes difficult the derivation of consensus nucleosome maps, but also it just makes impossible the development of predictive models to explain nucleosome positioning.

Discrepancy between nucleosome maps may originate from different sources: (i) the experimental conditions (such as MNase digestion levels or sequencing protocol); (ii) data processing for nucleosome calling; (iii) heterogeneity of the samples, derived from diversity of cellular states in the culture, such as cycle phase; (iv) variations among the samples derived from differences in the growth media (such as pheromones, stress, etc.) and (v) nucleosome dynamics across the genome, that will be detected as positional ‘fuzziness’ in the experimental nucleosomal (48,49).

We present here a systematic analysis of the effect of noise and nucleosome dynamics in defining nucleosome profiles in *Saccharomyces cerevisae*. We have been able to reduce cell-to-cell variability and cell cycle-induced nucleosome dynamics by using carefully synchronized cells and compare them against unsynchronized control cells grown under the same basal conditions. We have then explored the potential sources of variability in nucleosome maps related to different MNase digestion levels (from very mild to very strong) and sequencing procedure (single- versus paired-end). Furthermore, to reduce potential uncertainties related to nucleosome calling algorithms, we have applied a single algorithm with standard defaults, in conjunction with analyses of nucleosome architecture, which are independent of the nucleosome calling algorithm.

Taken together, our findings have allowed us a comprehensive evaluation of nucleosome positioning and stability that may contribute to partially uncover the underlying principles of nucleosome architecture dynamics. The picture derived from our analyses presents navigating nucleosomes along one-dimensional string (i.e. the DNA fiber) that are mainly positioned at specific places in response to strong nucleosome depletion signals generated by either intrinsic properties of DNA, the presence of competing DNA-binding proteins or chromatin-remodeling systems. The proposed model allows a very simple integration of different nucleosome positioning models, and provides clues on the impact of nucleosome fuzziness in the modulation of gene activity.

## MATERIALS AND METHODS

### Cell-cycle synchronization

Yeast strain BY4741 was grown using fresh YPD media at 30°C until an OD_600_ of 0.2. Then, alpha-factor mating pheromone (GenScript) was added to the culture to a final concentration of 10 μM and the culture was incubated for 2 h to induce cell-cycle arrest in late G1. As a control, an asynchronous culture was grown in parallel to an OD_600_ of 0.8. Both G1-arrest synchronized and asynchronous samples were collected and washed twice with phosphate buffered saline (PBS).

Cell synchrony was monitored by three approaches: flow cytometry (FACS), fluorescence microscopy and budding index calculation. For FACS analysis, cells were fixed with 100% EtOH, spun down and washed once with 1× SSC buffer (150 mM NaCl, 15 mM sodium citrate, pH 7.80). Removal of RNA and proteins were carried out by incubation with RNase A (0, 5 mg/ml, Roche) and proteinase K (0.5 mg/ml, Roche), correspondingly. Samples were briefly sonicated by using the Bioruptor system and mixed with 500 µl SSC buffer containing 0.1 mg/ml propidium iodide (PI, Sigma-Aldrich). Fluorescence emitted from DNA-intercalated PI was measured by Beckman Coulter EPICS® XL flow cytometer.

Cell-cycle phase was also monitored by fluorescence microscopy and budding index calculation. For these purposes, cells were briefly sonicated and fixed with EtOH in a similar manner as for FACS. Fixed cells were then resuspended in 200 µl PBS containing Hoechst stain at 30 µg/ml. Finally, cells were placed on a glass slide and visualized by fluorescence microscopy (Nikon E600 microscope). For budding index calculation, a sample from EtOH-fixed cells was placed on a hemocytometer and visualized under a phase contrast microscope to count the number of budded and unbudded cells.

### Nucleosomal DNA extraction

Nucleosomal DNA from G1-arrest and asynchronous samples was prepared as previously described (15). The overall nucleosome digestion was accurately controlled by carrying out several digestion reactions with MNase at concentrations of 0.04, 0.08, 0.12 and 0.16 U, respectively, at 37°C for 30 min. The reactions were stopped by addition of EDTA to a final concentration of 0.02 M and subsequently incubated with RNase A (0.1 mg) for 1 h at 37°C and further treated with Proteinase K at 37°C for 1 h. DNA was extracted using phenol–chloroform extraction and concentrated by ethanol precipitation.

The percentage of mononucleosomal DNA fragments was examined by 2% agarose gels. Furthermore, the integrity and size distribution of digested fragments were determined using the microfluidics-based platform Bioanalyzer (Agilent) prior to DNA sequencing. Typically, samples containing >80% mononucleosomal fragments were sent for sequencing. In addition, over- and under-digested samples were selected based on the proportion of mononucleosomal fragments. Over-digested samples were obtained by 0.12 U MNase digestions, which yielded to only mononucleosomes. Under-digested samples were obtained by 0.04 U of MNase yielding to mono-, di- and tri-nucleosomes.

### Nucleosomal DNA sequencing

Libraries of nucleosome fragments were prepared and adapted for deep sequencing using the standard Illumina protocol and sequenced them as single-end paired-end on Genome Analyzer IIx and HiSeq 2000 devices. Data were processed with a standard GA base calling pipeline to convert initial raw images into sequences, as described previously (15). Raw reads are available at the ENA-SRA website (http://www.ebi.ac.uk/ena) with accession number ERP004019.

### RNA Isolation and gene-expression arrays

Cells were collected at the same interval as nucleosomal DNA samples in icy-water and harvested by spinning for 3–4 min at 6000 rpm, frozen in liquid nitrogen and stored at −80°C. Total cellular RNA was extracted using the RNeasy kit (Qiagen), following the manufacturer’s instructions with the spheroplasting protocol (0.5 mg/ml zymolase). The total RNA was hybridized to Affymetrix GeneChip Yeast Genome 2.0 arrays for gene-expression analysis. Raw and processed files available in ArrayExpress under accession number E-MTAB-2195.

### Data processing and nucleosome calling

Reads from all samples were mapped to yeast genome (SacCer3, UCSC) with Bowtie (50) aligner and imported in R/Bioconductor framework (51). Single-end reads were resized to 50 bp and shifted downstream to align reads mapping in opposite strands using nucleR (52). Paired-end reads were trimmed to 50 bp maintaining the original center. Genome-wide coverage was normalized using the total number of reads in every experiment and scaled by a factor of 10^6^ to obtain the units of reads per million (r.p.m.). Peak calling was performed after noise filtering using nucleR parameters: peak width = 125 bp, peak detection threshold = 35%, maximum overlap = 50 bp.

### TSS clustering

Using the nucleosome calls obtained previously, we classified every gene according to their nucleosome architecture around the TSS. The closest nucleosome at or immediately downstream TSS was annotated as the +1 nucleosome. The nucleosome immediately upstream of the +1 nucleosome was annotated as the −1 nucleosome. After a visual analysis of the classifications, nucleosome calls were considered as well-positioned (W) when nucleR peak width score (score_w; positioning) and height score (score_h; coverage) were higher than 0.4 and 0.6, respectively. Otherwise, the nucleosome call was considered fuzzy (F). Accordingly with previous observations (39), the NFR was defined as the distance between the dyads of the −1/+1 nucleosome and it was annotated as ‘open’ if this distance was >215 bp or as ‘closed’ otherwise. The classification of a given gene was determined by the positioning of the −1 nucleosome, the width of the NFR and the positioning of the +1 nucleosome. Special cases such as when the −1 nucleosome was >300 bp further from the TSS (annotated as M, missing), the −1/+1 nucleosome calls were overlapped or when the regions −300:+300 bp had more than a 25% of uncovered bases were excluded from the analysis.

### Evaluation of nucleosome architecture variability between genes

In order to obtain an accurate estimate of gene architecture similarity/dissimilarity between samples, we defined the following metrics. Profile: we considered a gene promoter stable between two samples if Pearson’s correlation in the window of −300:300 bp around TSS was >0.7; conversely, we consider variable if the correlation was <0.5. Cluster: we considered a gene promoter stable if cluster dimensions (–1/NFR/+1) matched; otherwise we annotated as a relevant variation of this architecture when two of the clustering dimensions varied between samples. The +1/–1 nucleosome: we considered a stable nucleosome classification if nucleR provided the same classification (with thresholds for score_w and score_h noted above) for the two samples; we considered a relevant change if the nucleosome call was changing in classification and the absolute difference of the aggregated score (nucleR’s default score = 0.5*score_h + 0.5*score_w) was >0.25 (which implies a change of at least one quartile of the classification). NFR: we considered it stable if the NFR distance was annotated equally between samples (open/close/overlap/missing); or variable when a change in class was happening and the distance between −1/+1 nucleosomes differed >100 bp. Those genes that do not satisfy any of the criteria are considered out of the stability/variability threshold.

### Elastic energy model

Elastic energy was calculated using a mesoscopic model of DNA flexibility (53–56) with parameters derived from molecular dynamics simulations (57) as described previously (15). In short, for every tiled sequence of 147 bp in the genome, we calculated the increment of energy required to pass from a relaxed DNA conformation to a nucleosome-shaped conformation, using an experimental reference structure (58).

### Statistical positioning model

The very simple statistical-positioning model featured in this article considers that, after the energetic barrier in the NFR, nucleosomes are arranged statistically with a lineal increasing fuzziness. We decided to simulate a population of nucleosome reads centered in the +1 nucleosome with a dyad deviation of 25 bp. Dyads of downstream nucleosomes (+2, +3, …) were spaced 147 + 14 bp (accounting for average linker DNA length) with an increasing deviation of the dyad of +5 bp in every step and a decreasing number of reads equal to the 4% of the previous peak. The dyad of the −1 nucleosome was placed 147+100 bp (247 bp in total) upstream the +1 for the closed NFR and 147+200 bp (347 bp in total) for the open NFR. The following upstream nucleosomes (–2, −3, …) were defined as in the case of the downstream model but adjusting the deviation in 35 bp in the −1 nucleosome plus 5 bp in every following step, with a linker length of 18 bp. Different values of the different parameters in the model were selected after a grid search maximizing the correlation of the model with the average experimental distribution.

### TFBS prediction

Transcription factor binding sites (TFBSs) were derived from the position weight matrices (PWMs) available in JASPAR database for yeast (59). For every PWM, the genome-wide binding scores and predicted TFBS were calculated using R/Bioconductor Biostrings library with default parameters. Regions with annotated TFBS were pooled and their coverage was calculated as a measure of global TF affinity genome-wide.

## RESULTS

In an attempt to eliminate the noise resulting from cell-population heterogeneity, we have synchronized yeast cultures at the late G1 cell-cycle phase. Two synchronized cultures (Supplementary Figure S1) were considered as biological replicas. We labeled them as replicas 1 and 2 and subsequently isolated their nucleosomal DNA under similar MNase-digestion conditions. The digestion level was determined visually by agarose gel electrophoresis and the microfluidics-based platform Bioanalyzer (Agilent), as shown in Supplementary Figure S2. Accordingly, both samples yielded a major peak ∼147 bp corresponding to mononucleosomes, a secondary defined peak ∼295 bp corresponding to di-nucleosomes and residual peaks at ∼60 bp that might be assigned to either tetrasomes or other DNA–protein complexes.

### The effect of single- versus paired-end sequencing

Eventually, replicas 1 and 2 were sequenced both as single-end (1x) and paired-end (2x), assuming a minimal sequencing bias. We obtained high sequencing read depths in all our datasets (fold-coverage ranges between 13X and 177X for individual experiments), which were then processed using nucleR package as described in methods (52). Nucleosome profiles around TSSs were classified based on the positioning of −1 nucleosome (fuzzy (F), well positioned (W) or missing (M)) and +1 nucleosome (F or W) according to their nucleR score (Materials and methods section), and the width of NFR. NFR state was identified as open (typically ∼130-bp wide) or closed (∼30-bp wide) according to previously reported bimodal distribution (39). We were able to classify ∼90% of yeast gene promoters into nucleosome architectures for both replicas (Supplementary Figure S3). The remaining 10% could not be classified due to either low coverage, undefined +1 or overlapping nucleosomes and were not considered in the remaining analysis. We explored the variability of the results based on different assignment criteria, and notably, nucleosome calls appeared quite robust regardless the threshold parameters applied in nucleR (Supplementary Table S1), proving the accuracy of the algorithm and further validating that detected variability does not respond to bioinformatics artifacts.

**Figure 1.**
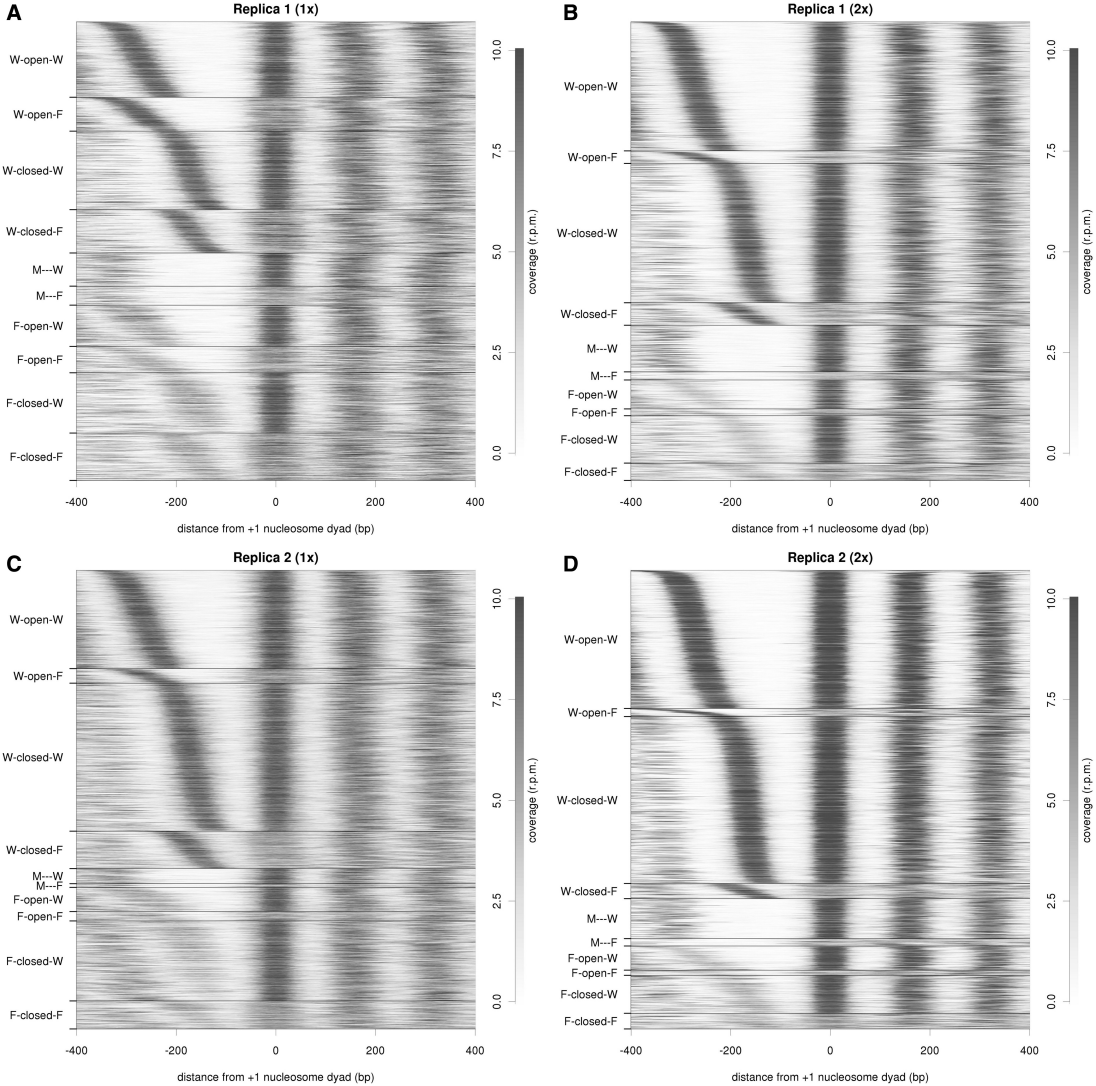
Nucleosome coverage and gene clustering in single- and paired-end sequencing. Heat maps showing nucleosome occupancy around TSS in replicas 1 (top) and 2 (bottom) for single-end sequencing (1x, left) and paired-end sequencing (2x, right). Genes are clustered based on their nucleosome profile and their coverage is plotted taking +1 nucleosome dyad as ′0′.

In principle, since every sample was both single- and paired-end sequenced, nucleosome maps should strongly resemble one another. However, when we compared the nucleosome profiles around TSSs, they displayed different architectures depending on the sequencing method (Figure 1, comparison of A and C with B and D). Single-end sequencing generated noisier nucleosome maps, leading to higher populations of fuzzy nucleosomes. This observation is illustrated in more detail in Supplementary Table S2, which shows the distribution of genes according to classifying parameters such as −1/+1 positioning or NFR width. Conversely, the percentage of −1 or +1 fuzzy nucleosomes is considerably reduced in paired-end sequenced samples, confirming that single-end sequencing yields an artificial enrichment of fuzzy nucleosomes. Interestingly, while the general coverage around TSSs is reasonably preserved (Supplementary Table S3 first column), only ∼38–36% of the nucleosome architectures are conserved (Supplementary Table S3, second column) and ∼23%of the genes show clearly distinct nucleosome classifications depending on the sequencing (1x versus 2x) method (Supplementary Table S3, seventh column). Intriguingly, when we analyzed the individual parameters describing a nucleosomal architecture, we observed that 63–64% of +1 nucleosome positions have the same annotation in both single- and paired-end sequencing, and only 5–7% display very different annotations. In the case of −1 nucleosome position, the preservation is less pronounced, with 61% conservation (52% for replica 2) and 7% discrepancy (12% for replica 2). Moreover 69% (56% in replica 2) of the NFRs maintain the same annotation, while 6%–9% can show width changes of up to >100 bp.

### The effect of biological replica variability

In order to minimize the noise coming from experimental protocols (out of the specific sequencing procedure), we compared the nucleosome maps of the two synchronized replicas generated by paired-end sequencing, which both present a similar coverage of 50X. As shown in Figure 1, nucleosome patterns are quite similar in both replicas, dominated by WoW, WcW and a myriad of families characterized by a +1 W and −1 F/M (Figure 1, Supplementary Figure S3). However, when we analyzed individual genes (Supplementary Table S3), significant differences arise between replicas. Nearly 90% of genes show reasonably similar coverage profiles, but only 48% of them maintain their nucleosome architecture in the two replicas. Even though the majority of changes are subtle, usually only affecting one nucleosome position (e.g. W→F or F→M), ∼15% of genes show significant changes in class annotation. Interestingly, only 3% of genes show dramatic changes in NFR in contrast to 6–7% that show different −1 and +1 nucleosome localizations (Supplementary Table S3), indicating that NFR is more conserved and less prone to variations than flanking nucleosome positions.

Taken together, our findings show that inter-replica variations are not negligible and point out that nucleosome positioning is intrinsically highly plastic and dynamic. In accordance with this observation, elastic energy models propose that a 10-bp sliding of a nucleosome would face a general energy barrier (i.e. the difference between the best and worse wrapping configuration) of ∼13 kcal/mol in average (<1 kcal/mol for sliding one single position), while in a larger scale, we would find mesoscopic barriers of ∼47 kcal/mol (Supplementary Figure S4), which might contribute to phasing. Similarly, atomistic molecular dynamics simulations detect spontaneous sliding of one base step at the multi-nanosecond time scale (60). Consequently, with such a dynamic organization, nucleosomes tend to change positions constantly and hence, well-positioned nucleosomes might not actually be intrinsically tightly placed in the absence of negative ‘nucleosome depletion’ signals. Indeed, the deformation energy required to wrap fuzzy or well-positioned nucleosomes is similar (∼200 kcal/mol for a 145-bp double-stranded DNA, Supplementary Figure S5). Fuzziness is then likely to be the default state for nucleosomes in a random DNA in the absence of additional factors, such as NFR.

### The effect of cell diversity

In order to determine the variability caused by cell heterogeneity, we included an asynchronous yeast culture in our analysis, labeled as ‘asynchronous’ sample (Supplementary Figure S1). This sample also produced well-defined nucleosomal maps, where 94% of yeast genes had well-located +1 nucleosomes and around 87% of promoters could be assigned to a particular nucleosomal architecture around TSS.

**Figure 2.**
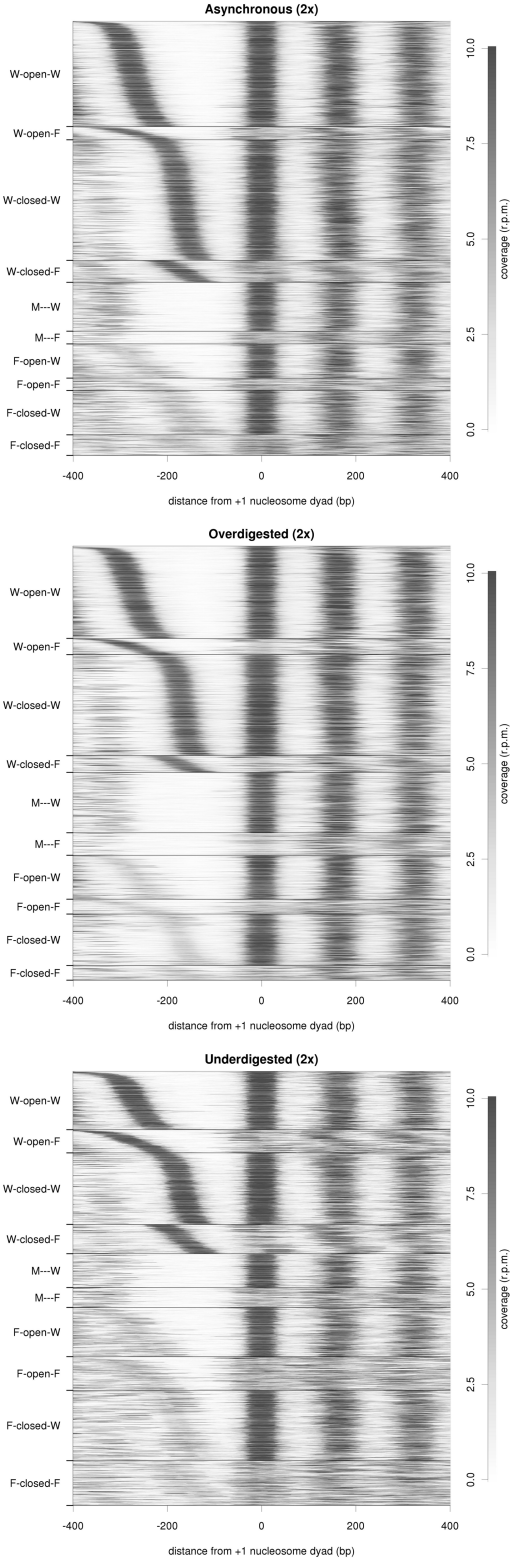
Nucleosome coverage and clustering under different experimental conditions. Similar to Figure 2, but for asynchronous (top), over-digested (middle) and under-digested (bottom) samples.

The asynchronous sample shows clear differences when compared to the synchronized replicas 1 and 2, as shown in Figures 1 and 2 and Supplementary Table S1. WoW and WcW nucleosome classes decrease while −1 F/M or +1 F nucleosome positions are more prevalent in asynchronous maps (proportion test *P*-value < 2.2 × 10^−^^16^), suggesting that cell-cycle progression may induce chromatin rearrangements around TSSs that may be reflected as diffuse nucleosome signals in MNase-Seq experiments derived from asynchronous samples.

Interestingly, analysis of individual genes provides a clearer impact of cell heterogeneity-dependent variability. Differences in nucleosome coverage profiles and nucleosome architectures are higher when the asynchronous sample is compared against biological replicas than when biological replicas are directly compared (Supplementary Table S2, columns 6 and 7). As anticipated from Figure 2, the increase in −1 and +1 nucleosome fuzziness seems to be the main responsible for variations in chromatin structure around TSSs (Supplementary Tables S1 and S2). In average 475 genes pass from WoW or WcW in rep1/2 to some fuzzy structure in the asynchronous replica. As a result the ratio well-positioned/non-well-positioned −1 nucleosomes decreases from 1.8–2.3 (replicas 1 and 2) to 1.4 (in asynchronous) (proportion test for replica 1 *P*-value = 1.07 × 10^−^^10^, for replica 2 *P*-value < 2.2 × 10^−16^), and similarly in case of +1 nucleosomes, going from 5.0–7.0 (replicas 1 and 2) to 3.7 (proportion test for replica 1 *P*-value = 5 × 10^−11^, for replica 2 *P*-value < 2.2 × 10^−^^16^). Conversely, NFR width between synchronized and asynchronous samples shows similar changes as between biological replicas, suggesting that cell cycle-dependent chromatin rearrangements do not lead to massive nucleosome eviction around TSSs, which would dramatically alter NFR dimensions (proportion test for replica 1 *P*-value = 0.22, for replica 2 *P*-value = 0.09).

Overall, our detailed comparison of cell cycle synchronized and asynchronous samples reveals that asynchronous experiments contain an additional source of noise due to the cell cycle-dependent nucleosome dynamics, and that caution is necessary with maps derived from asynchronous samples (those typically available in the literature), since average maps can mask the existence of two populations with completely different nucleosomal architectures. Fuzzy nucleosome signals can be in reality the result of a mixed population where some cells contain well-localized nucleosomes while others are nucleosome-depleted at the same region, or rather that a well-localized nucleosome has moved to a neighboring position in different cells (Supplementary Figure S6). Indeed, we observed that 105 genes displayed very similar coverage profiles around TSSs between biological replicas (Pearson’s correlation > 0.7), but clearly differed with the asynchronous sample (Pearson’s correlation < 0.5). Of note, although GO and pathway annotation analyses were not able to find any particular enrichment in this set of genes, 15 of them are annotated as cell cycle periodic genes in Cyclebase (61), which represents a small enrichment in cell-cycle related functions (from 9.8% in genomic mean to 14%; proportion test *P*-value = 0.087). Interestingly, cell cycle-related genes belong to the G1/S regulon rather than to the alpha-factor pathway, indicating that the source of variation does not derive from alpha-factor stimuli. A more detailed analysis would require data for every individual cell stages, but nonetheless, the present results support the notion that chromatin reorganization might be coupled to cell-cycle regulation mechanisms.

### The effect of MNase digestion

To investigate the bias effect of MNase digestion on the generation of nucleosome maps (a quite ignored source of variability), we have used two additional MNase-Seq experiments derived from a G1-synchronized culture but treated under either more aggressive (over-digested sample) or milder (under-digested sample) MNase-digestion conditions. The samples were sequenced using paired-end technology and a similar data processing for a direct comparison with other replicas.

As shown in the Bioanalyzer histograms (Supplementary Figure S2), over-digestion of chromatin leads to the disappearance of the di-nucleosome signal and to a broader mono-nucleosome peak that is shifted towards shorter fragments, probably caused by certain intra-nucleosomal cleavage. Nucleosome architectures of the over-digested sample are well-defined, with a clear +1 nucleosome signal in 95% of the gene promoters and unambiguously assigned nucleosome families, similar to replicas 1 and 2. Yet, the analysis of nucleosome pattern distributions reveals clear differences between the over-digested sample and replicas 1 and 2 (Figure 2, Supplementary Table S2). In over-digested chromatin, the prevalence of canonical nucleosome classes (i.e. WoW and WcW) decreases and fuzzy −1 nucleosomes are enriched. Thus, ∼800 genes move from WoW/WcW patterns in replicas 1 and 2 to a fuzzier configuration in the over-digested sample (proportion test *P*-value < 2.2 × 10^−^^16^). The number of missing −1 nucleosomes increases from 711–510 (replicas 1 and 2) to 1154 (proportion test *P*-value < 2.2 × 10^−16^). Overall, these findings suggest that excessive MNase digestion can lead to partial degradation of some well-positioned nucleosomes, resulting in fuzzier nucleosome peaks, or even to the complete disassociation of unstable nucleosomes, leading to loss of certain nucleosome signals. This behavior is further confirmed with a higher mean deviation of the nucleosome dyad position in the over-digested sample (Kolmogorov–Smirnov test *P*-value = 2·10^−^^6^) (Supplementary Figure S7).

We have further explored the impact of over-digestion on nucleosomal architectures by the analysis of individual genes (Supplementary Table S3). While only 15% of the genes show clearly different nucleosome arrangements between replicas, up to 25% (18% in replica 2) change with respect to the over-digested sample. Similarly, the variability in −1 and +1 nucleosome annotations also increases from 6–7% to 10–13%. In contrast, NFR width seems to be very resistant to digestion conditions, pointing out that a more aggressive digestion mostly result in partial degradation of nucleosomes but rarely in their complete eviction around TSSs (Supplementary Tables S2–S3 and Figure 2).

On the other hand, the under-digested sample exhibits well-defined mono-, di-, tri- and even tetra-nucleosomal peaks (Supplementary Figure S2). Intriguingly, paired-end sequencing only yielded 78% of gene coverage and ∼69% of TSSs could be classified into nucleosome families, whereas the classification was 86.5 ± 3.7% in previous experiments. The significantly (proportion test *P*-value = 8 × 10^−^^9^ for replica 1, *P*-value = 2 × 10^−16^ for replica 2) enrichment of depleted areas might account for the under-representation of longer fragments in the sequencing reactions, since it is well established that deep sequencing favors the amplification of short over long fragments (62). Moreover, we observed a higher frequency of overlapping nucleosomes (10.2% in over-digested in comparison with 4.6% and 2.5% in replicas 1 and 2, proportion test *P*-value < 2.2 × 10^−^^16^), which might correspond in reality to DNA in complex with other proteins. Interestingly, the uncovered regions specific to under-digested sample are distributed over the entire genome without any significant enrichment according to GO analysis. Yet, they show a clear preference for AT-rich segments (3.38 higher fold) and intergenic regions (15% enrichment over background, simulated *P*-value < 10^−^^5^).

**Figure 3.**
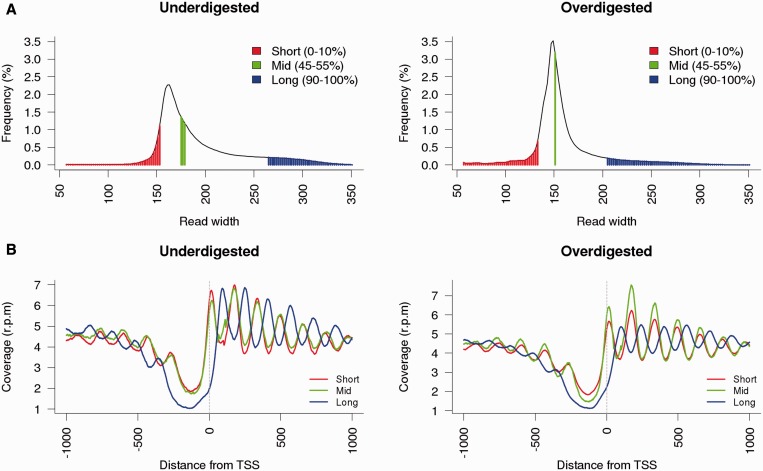
Effect of variable read length on map coverage. (**A**) Coverage distribution of short, mid and long reads in under-digested (left) and over-digested (right) samples. (**B**) Normalized coverage profiles of trimmed reads around TSSs derived from different sequencing lengths.

Despite the poor ability of sequencing protocols to deal with long fragments, we were able to recover several long reads analogous to di-nucleosomal signals, being ∼3% of them longer than 300 bp in the under-digested sample. In contrast, we only rescued 0.4% of these long reads in the over-digested sample. Therefore, the presence of di-nucleosome signals introduce another source of noise in the nucleosome maps, since nucleosome calling algorithms usually consider peak signals as mono-nucleosomes and align them on the genome based on their middle position, which is assumed to correspond to the nucleosome dyad. However, the mid-point of di-nucleosomes is actually located on the linker region, leading to a counter-phase location with respect to mono-nucleosome signals. This observation is shown in Figure 3, where short-, mid- or long-sized fragments of over- and under-digested samples are compared. The counter-phasing is more explicit in under-digestion, which contains more di-nucleosome derived signals and thus, leads to a higher noise in terms of linker length and nucleosome phasing (Figure 3B).

Overall, our observations show that MNase digestion levels may strongly bias nucleosome maps by intra-nucleosomal cleavage or the introduction of longer internucleosomal or non-nucleosomal protected regions. Caution is then necessary, since MNase is not a mere spectator of nucleosome architecture, but bias it in one way or the other, introducing a noise that need to be considered when maps obtained under different degradation conditions are compared. This warning is especially important since MNase is an enzyme whose activity is not always easy to control.

### The effect of sequencing read depth

Interestingly, when comparing paired-end experiments with extremely large read depth (for example over-digested (2x) and asyncronized (2x) datasets, with a coverage of 146X and 177X, respectively), the nucleosomal maps do not show better agreement than when comparing datasets with lower read depth (such as single-end replicas 1 and 2, with coverage ∼13X and 45X, respectively). Certainly, low read-depth can increase the noise in nucleosome maps but it does not seem to be a dramatic contributor in the present article.

### The effect of basal expression level

In order to assess the possible effect of noise due to different transcription rates, we measured the absolute levels of gene expression in our G1 synchronized samples (replicas 1 and 2) using expression arrays (see Materials and methods section). Based on the hybridization results, we then selected the top 500 and the bottom 500 genes according to their normalized expression level and analyzed in detail the nucleosome organization around TSSs. Lowly expressed genes display better defined nucleosome organization than highly expressed genes (with ∼8% more robust coverage profiles; Wilcoxon rank sum test *P*-value < 0.0002). Notably, this differential organization is increased to 13% when gene body is considered (Wilcoxon rank sum test *P*-value < 2.2*e* – 16).

### Underlying factors in nucleosome positioning

Heterogeneity in MNase-Seq experiments may be the responsible of the low accuracy in the available nucleosome positioning predictive models when other than the training datasets are employed, challenging the validation of nucleosome positioning models. In contrast, our systematic analysis under controlled conditions allowed us to obtain a robust set of nucleosome profiles (showing a correlation >0.7 and displaying the same nucleosome architecture in biological replicas). This set (comprising 3096 genes) represents the well-conserved nucleosome architectures in late G1 cell-cycle phase and reveals that WcW (1306 genes) and WoW (1164 genes) are the most pervasive classes, followed by M–W (263 genes) and FcF (155 genes) classes. We can use then these maps to evaluate the goodness of different predictive models, without being exposed to noise and uncertainties in the experimental data.

**Figure 4.**
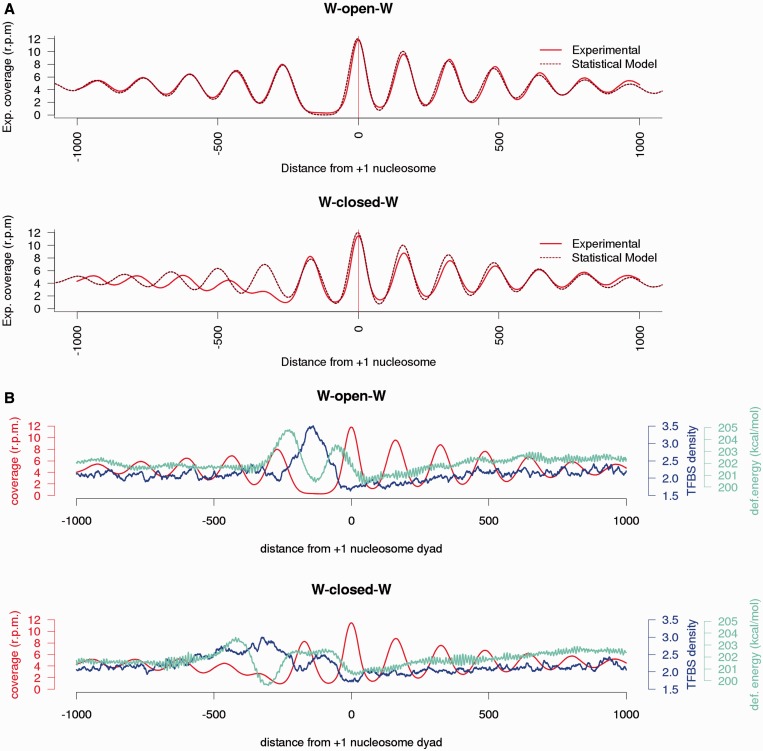
Statistical positioning and intrinsic DNA energetic barriers. (**A**) Average experimental nucleosome coverage from WoW (top) and WcW (bottom) patterns in replica 1 are compared against a nucleosome positioning statistical model. (**B**) The experimental coverage of WoW (top) and WcW (bottom) classes (red) are overlapped with deformation energy (cyan) and predictive TFBS (blue) around TSSs.

We compared the experimental WcW and WoW nucleosome maps with an extremely simple statistical model (see Materials and methods section) that places nucleosomes every 161–165 bp (147 + 14 – 18 bp due to linker variation), starting from NFR with decreasing nucleosome phasing (Figure 4A). Once the NFR is defined, the model is able to explain the majority of nucleosome positioning around TSSs, further confirming that nucleosome location in TSSs is largely determined by a barrier, i.e. NFR, which subsequently places arrays of nucleosomes with decay in positioning as they separate from the NFR signal. It seems then that the crucial step to position nucleosomes is to determine the placement of the NFR. To analyze the determinants of NFR position we studied TFBS and compute the deformation energy required to wrap DNA around the histone core (see ‘Materials and Methods' section). As shown in Figure 4B, NFRs at WoW architectures present intrinsically different DNA properties leading to an anomalously large deformation energies at NFR. This suggests that physical properties can define the boundaries off the NFRs in this family, but they also erroneously predict a well-positioned nucleosome in the middle of the NFR. It is clear (Figure 4B) that competition with TFs avoids the binding of the nucleosome in the middle of the NFR. Clearly, TF binding is then crucial in determining the integrity of NFRs and hence the phasing of the nucleosome arrays in WoW architectures. The synergetic effect of physical properties and TFBS is also clear in nucleosome placements in the WcW family, where the region around TSS is marked by an unusual profile of physical properties and a distinct pattern of TFBSs. The strong +1 nucleosome signal fits perfectly in a region of low cost for wrapping DNA around a nucleosome and depleted in TFBS.

Taken together, our analysis of robust nucleosome maps suggest that simple statistical positioning is a key responsible of the basal nucleosome positioning, with NFR being the main signal organizing nucleosome string. Physical properties and binding to effector proteins, such as TFs, act in a synergistic manner to define NFR location and boundaries and to define then the phasing of the nucleosome string. Clearly, the chromatin remodelers will act on this basal activity to facilitate positioning of nucleosomes in a regularly spaced array.

Lastly, we analyzed the heterogeneity in nucleosome architecture along coding regions, taking into account that they do not show the typical TSS nucleosome pattern (see Supplementary Tables S4–S5). Interestingly, our findings indicate that the conclusions obtained for TSS regions are also valid for gene body regions. Clearly, the heterogeneity and plasticity in nucleosome architecture is not a differential property of the TSS vicinities, since also coding regions show a significant degree of flexibility that surely reflects the intrinsic mobility of nucleosomes, especially for highly expressed genes.

## DISCUSSION

Many genome-wide nucleosome maps are now available in the literature for model organisms, but the correlation among the aligned maps is typically poor. This variability is a first indication that nucleosomes are not as rigidly placed as suggested from single dataset analyses. However, it is difficult to determine at what extent such variability is due to biological sources (for instance action of chromatin remodelers) or related to experimental procedures (such as MNase different activity rate).

**Figure 5.**
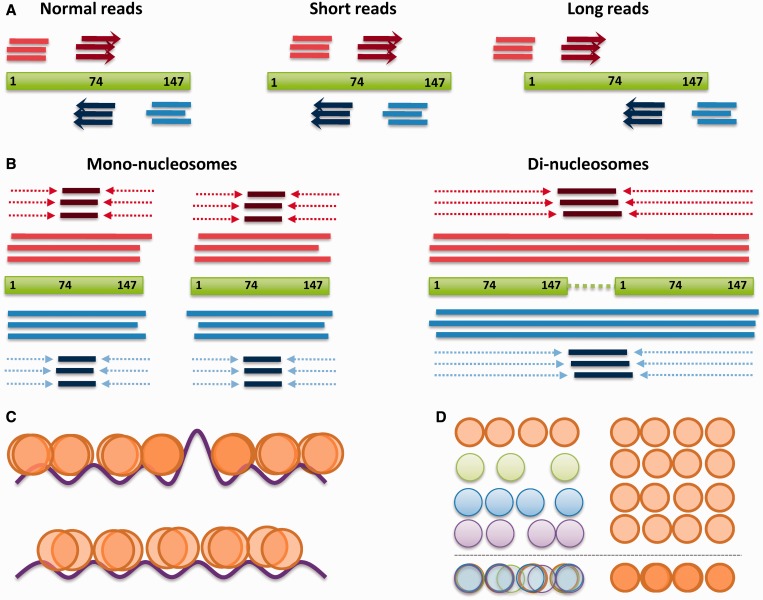
Possible sources of intrinsic nucleosome noise. (**A**) In single-end sequencing, reads mapped in opposite strands (light red, light blue) are shifted 74-bp downstream to align the nucleosome dyad (dark red, dark blue). Despite this approach is suitable for mono-nucleosome fragment alignment (left), shorter (middle) or longer fragments (right) are misaligned, causing a fuzzy peak coverage. (**B**) In paired-end sequencing, the detection of mono-nucleosome dyads can be obtained by trimming the reads (left). However, in the case of long di-nucleosome fragments, the trimmed reads are aligned to the linker space between mono-nucleosomes, which in turn increase the fuzziness. (**C**) Energetic barriers due to intrinsic DNA deformability potential or presence of competing proteins (represented as purple line) act as a phasing element in adjacent nucleosomes (top) leading to well-localized nucleosome signals. In the absence of such barriers, the periodicity of this potential cannot act in nucleosome phasing (bottom) leading to diffuse signals originated by spontaneous nucleosome sliding. (**D**) Individual nucleosome arrays of asynchronous cells in different stages of the cell-cycle capture intrinsic chromatin dynamics (left) which is visualized as fuzzy signals. This effect is minimized in synchronized cell populations (right).

In an attempt to remove, as much as possible, experimental and sequencing biases, we have addressed several sources of noise that might lead to artifactual nucleosome fuzziness in MNase-Seq derived nucleosome maps (Figure 5). Single- and paired-end sequencing of identical samples demonstrates that single-end sequencing generates a high level of fuzziness, prompting to low reproducible nucleosome maps and challenging accurate analyses of nucleosome arrangements along the DNA fiber. Furthermore, when identical samples are treated under different MNase digestion conditions, high MNase levels lead to intra-nucleosomal cleavage and partial degradation of some well-positioned, but not necessarily very stable, nucleosomes. Since the two ends of the nucleosome might not be equally protected due to differences in DNA–histone interactions (63,64), over-digestion can lead to unsymmetrical degradation of nucleosome, which could be reflected in nucleosome maps as an increase in the overall fuzziness. On the other hand, milder digestion can also lead to severe noise, since the poor sequencing of long reads might cause loss of information about those regions enriched in long fragments. Moreover, the alignment of di-nucleosomal signals can yield to counter-phased positioning with respect to mono-nucleosomal signals, generating noise in the nucleosome maps, especially in the linker regions.

Computational processing of raw data is another source of ‘spurious noise’. Converting read-alignment information into nucleosome positions implies the assumption of some predictive models (especially for single-end sequencing reads). Furthermore, the classification of nucleosome signals also requires the use of arbitrary thresholds. We have minimized these potential factors by applying various classification thresholds in the nucleR nucleosome calling algorithm and by the use of orthogonal metrics such as direct read information. Our findings suggest that the detected variability in our analyses do not respond to computational artifacts. Additionally, the observed variability is not a consequence of poor sequencing coverage either, since no reduction of variability is found when samples with relatively high and low coverage are compared.

Up to date, most of the available MNase-Seq derived nucleosome maps originate from asynchronous populations. This asynchrony can produce considerable amount of noise due to cell cycle-dependent nucleosome changes which may be related to gene-expression alterations or chromatin compaction and relaxation. Therefore these maps actually reproduce average nucleosome phasing in different populations, some of which might be stable and well-positioned in one cell while more diffuse in others. Such variations can generate misleading nucleosome maps that might not accurately reproduce the actual chromatin organization, and in fact only reflect the statistical averaging of different cell populations. Consistently, our comparative analysis between synchronized and asynchronous samples shows that asynchrony contributes to an increase of ∼30% in nucleosome fuzziness. Regions with striking chromatin alterations along cell cycle are usually detected as regions with fuzzy and not well-defined nucleosomes in the usual asynchronous maps. Interestingly, highly active genes in a certain cell-cycle stage show in general less-conserved nucleosome distributions, connecting gene activity and nucleosome mobility.

We have tried to further minimize the biological noise level by generating nucleosome maps from cell cycle synchronized biological replicas, in order to have a reliable detection and accurate analysis of nucleosome positioning. Although average nucleosome maps are identical, differences at the gene level are not negligible. Thus, even if general nucleosome profiles are reasonably conserved, only 30% of the well-positioned nucleosomes are located at the same genomic place (within ±5-bp deviation) in two biologically equivalent replicas. Our results suggest that in principle nucleosomes are mobile along the DNA fiber, since intrinsic sliding barriers are small. This can lead to a kind of ‘spread’ of signal along average position, or alternatively, to the population of different nucleosomal arrangements in different cells, giving in all cases to a fuzzy signal in nucleosomal maps.

The localization pattern that we (and many others before) have found obeys at great extent to a simple statistical positioning, where nucleosome arrays are placed starting from NFRs (which are defined with a small noise level and a high reliability in biological replicas), with a lineal decrease in positioning as separated from the NFR. NFR borders are marked by unusual DNA physical properties that hampers wrapping of DNA around histone core (*in vitro* and *in vivo*) and by regions which are highly prevalent in TFBSs (*in vivo*). Under normal physiological conditions, sequence-encoded physical properties and protein binding (including TFBSs) act synergistically to define the NFR and accordingly, the nucleosome array. Such synergy can be enhanced or reduced by chromatin remodelers, which are shown to facilitate nucleosome positioning by packing them against a barrier, like NFRs (18,31). This could partially explain the differences often found between *in vivo* and *in vitro* nucleosomal maps.

Taken together, our robust set of nucleosome profiles have enabled us to carefully inspect the various sources of noise and dissociate them from the actual nucleosome dynamics in the cell, which in all cases are otherwise captured as positional ‘fuzziness’ in the nucleosome maps. Finally, our systematic approach provides an insight into nucleosome positioning determinants and may guide the standardization of MNase-Seq experiments in order to generate reproducible genome-wide nucleosome patterns. Finally, our results shed light in the requirement of discarding the current static picture of nucleosome positioning and start considering nucleosomes as intrinsically mobile entities navigating along the DNA fiber.

## ACCESSION NUMBERS

Raw reads are available at the ENA-SRA website (http://www.ebi.ac.uk/ena) with accession number ERP004019. Raw and processed expression data for G1 stage is available in ArrayExpress platform under accession number E-MTAB-2195.

## SUPPLEMENTARY DATA

Supplementary Data are available in at NAR Online.

## FUNDING

Spanish Ministry of Science and Innovation [BIO2012-32868]; Instituto de Salud Carlos III (INB); European Research Council (SimDNA project). Funding for open access charge: Spanish Ministry of Science and Innovation [BIO2009-10964 and Consolider E-Science], Instituto de Salud Carlos III (INB-Genoma España and COMBIOMED RETICS) and Fundación Marcelino Botín.

*Conflict of interest statement*. The funders had no role in study design, data collection and analysis, decision to publish, or preparation of the manuscript.

## Supplementary Material

Supplementary Data

## References

[1] Kornberg RD, Lorch Y (1999). Twenty-five years of the nucleosome, review fundamental particle of the eukaryote chromosome. Cell.

[2] Malik HS, Henikoff S (2003). Phylogenomics of the nucleosome. Nat. Struct. Biol..

[3] Luger K, Mäder AW, Richmond RK, Sargent DF, Richmond TJ (1997). Crystal structure of the nucleosome core particle at 2.8 A resolution. Nature.

[4] Tolstorukov MY, Volfovsky N, Stephens RM, Park PJ (2011). Impact of chromatin structure on sequence variability in the human genome. Nat. Struct. Mol. Biol..

[5] Tilgner H, Nikolaou C, Althammer S, Sammeth M, Beato M, Valcárcel J, Guigó R (2009). Nucleosome positioning as a determinant of exon recognition. Nat. Struct. Mol. Biol..

[6] Workman JL, Buchman AR (1993). Multiple functions of nucleosomes and regulatory factors in transcription. Trends Biochem. Sci..

[7] Jiang C, Pugh BF (2009). A compiled and systematic reference map of nucleosome positions across the Saccharomyces cerevisiae genome. Genome Biol..

[8] Kaplan N, Moore IK, Fondufe-Mittendorf Y, Gossett AJ, Tillo D, Field Y, LeProust EM, Hughes TR, Lieb JD, Widom J (2009). The DNA-encoded nucleosome organization of a eukaryotic genome. Nature.

[9] Lee C-K, Shibata Y, Rao B, Strahl BD, Lieb JD (2004). Evidence for nucleosome depletion at active regulatory regions genome-wide. Nat. Genet..

[10] Lee W, Tillo D, Bray N, Morse RH, Davis RW, Hughes TR, Nislow C (2007). A high-resolution atlas of nucleosome occupancy in yeast. Nat. Genet..

[11] Sadeh R, Allis CD (2011). Genome-wide “re”-modeling of nucleosome positions. Cell.

[12] Segal E, Fondufe-Mittendorf Y, Chen L, Thåström A, Field Y, Moore IK, Wang J-PZ, Widom J (2006). A genomic code for nucleosome positioning. Nature.

[13] Zhang L, Ma H, Pugh BF (2011). Stable and dynamic nucleosome states during a meiotic developmental process. Genome Res..

[14] Albert I, Mavrich TN, Tomsho LP, Qi J, Zanton SJ, Schuster SC, Pugh BF (2007). Translational and rotational settings of H2A.Z nucleosomes across the Saccharomyces cerevisiae genome. Nature.

[15] Deniz Ö, Flores O, Battistini F, Perez A, Soler-Lopez M, Orozco M (2011). Physical properties of naked DNA influence nucleosome positioning and correlate with transcription start and termination sites in yeast. BMC Genomics.

[16] Rach EA, Winter DR, Benjamin AM, Corcoran DL, Ni T, Zhu J, Ohler U (2011). Transcription initiation patterns indicate divergent strategies for gene regulation at the chromatin level. PLoS Genet..

[17] Radman-Livaja M, Verzijlbergen KF, Weiner A, van Welsem T, Friedman N, Rando OJ, van Leeuwen F (2011). Patterns and mechanisms of ancestral histone protein inheritance in budding yeast. PLoS Biol..

[18] Zhang Z, Wippo CJ, Wal M, Ward E, Korber P, Pugh BF (2011). A packing mechanism for nucleosome organization reconstituted across a eukaryotic genome. Science.

[19] Wang X, Bai L, Bryant GO, Ptashne M (2011). Nucleosomes and the accessibility problem. Trends Genet..

[20] Kornberg R (1981). The location of nucleosomes in chromatin: specific or statistical?. Nature.

[21] Mavrich TN, Ioshikhes IP, Venters BJ, Jiang C, Tomsho LP, Qi J, Schuster SC, Albert I, Pugh BF (2008). A barrier nucleosome model for statistical positioning of nucleosomes throughout the yeast genome. Genome Res..

[22] Field Y, Kaplan N, Fondufe-Mittendorf Y, Moore IK, Sharon E, Lubling Y, Widom J, Segal E (2008). Distinct modes of regulation by chromatin encoded through nucleosome positioning signals. PLoS Comput. Biol..

[23] Miele V, Vaillant C, D’Aubenton-Carafa Y, Thermes C, Grange T (2008). DNA physical properties determine nucleosome occupancy from yeast to fly. Nucleic Acids Res..

[24] Morozov AV, Fortney K, Gaykalova DA, Studitsky VM, Widom J, Siggia ED (2009). Using DNA mechanics to predict in vitro nucleosome positions and formation energies. Nucleic Acids Res..

[25] Lantermann AB, Straub T, Strålfors A, Yuan G-C, Ekwall K, Korber P (2010). Schizosaccharomyces pombe genome-wide nucleosome mapping reveals positioning mechanisms distinct from those of *Saccharomyces cerevisiae*. Nat. Struct. Mol. Biol..

[26] Ioshikhes I, Hosid S, Pugh F (2011). Variety of genomic DNA patterns for nucleosome positioning. Genome Res..

[27] Scipioni A, De Santis P (2011). Predicting Nucleosome Positioning in Genomes: Physical and Bioinformatic Approaches. Biophys. Chem..

[28] Trifonov EN (2011). Cracking the chromatin code: Precise rule of nucleosome positioning. Phys. Life Rev..

[29] Zhang Y, Moqtaderi Z, Rattner BP, Euskirchen G, Snyder M, Kadonaga JT, Liu XS, Struhl K (2009). Intrinsic histone-DNA interactions are not the major determinant of nucleosome positions in vivo. Nat. Struct. Mol. Biol..

[30] Valouev A, Johnson SM, Boyd SD, Smith CL, Fire AZ, Sidow A (2011). Determinants of nucleosome organization in primary human cells. Nature.

[31] Yen K, Vinayachandran V, Batta K, Koerber RT, Pugh BF (2012). Genome-wide nucleosome specificity and directionality of chromatin remodelers. Cell.

[32] Struhl K, Segal E (2013). Determinants of nucleosome positioning. Nat. Struct. Mol. Biol..

[33] Tsankov A, Yanagisawa Y, Rhind N, Regev A, Rando OJ (2011). Evolutionary divergence of intrinsic and trans-regulated nucleosome positioning sequences reveals plastic rules for chromatin organization. Genome Res..

[34] Wang X, Bryant GO, Floer M, Spagna D, Ptashne M (2011). An effect of DNA sequence on nucleosome occupancy and removal. Nat. Struct. Mol. Biol..

[35] Hughes AL, Jin Y, Rando OJ, Struhl K (2012). A functional evolutionary approach to identify determinants of nucleosome positioning: a unifying model for establishing the genome-wide pattern. Mol. Cell.

[36] Segal E, Widom J (2009). What controls nucleosome positions?. Trends Genet..

[37] Hihara S, Pack C-G, Kaizu K, Tani T, Hanafusa T, Nozaki T, Takemoto S, Yoshimi T, Yokota H, Imamoto N (2012). Local nucleosome dynamics facilitate chromatin accessibility in living mammalian cells. Cell Rep..

[38] Möbius W, Osberg B, Tsankov AM, Rando OJ, Gerland U (2013). Toward a unified physical model of nucleosome patterns flanking transcription start sites. Proc. Natl Acad. Sci. USA.

[39] Zaugg JB, Luscombe NM (2011). A genomic model of condition-specific nucleosome behaviour explains transcriptional activity in yeast. Genome Res..

[40] Vavouri T, Lehner B (2011). Chromatin organization in sperm may be the major functional consequence of base composition variation in the human genome. PLoS Genet..

[41] Arya G, Maitra A, Grigoryev SA (2010). A structural perspective on the where, how, why, and what of nucleosome positioning. J. Biomol. Struct. Dyn..

[42] Schones DE, Cui K, Cuddapah S, Roh T-Y, Barski A, Wang Z, Wei G, Zhao K (2008). Dynamic regulation of nucleosome positioning in the human genome. Cell.

[43] Huebert DJ, Kuan P-F, Keleş S, Gasch AP (2012). Dynamic changes in nucleosome occupancy are not predictive of gene expression dynamics but are linked to transcription and chromatin regulators. Mol. Cell. Biol..

[44] Bai L, Morozov AV (2010). Gene regulation by nucleosome positioning. Trends Genet..

[45] Kuan PF, Huebert D, Gasch A, Keles S (2009). A non-homogeneous hidden-state model on first order differences for automatic detection of nucleosome positions. Stat. Appl. Genet. Mol. Biol..

[46] Tsankov AM, Thompson DA, Socha A, Regev A, Rando OJ (2010). The role of nucleosome positioning in the evolution of gene regulation. PLoS Biol..

[47] Ozonov EA, van Nimwegen E (2013). Nucleosome free regions in yeast promoters result from competitive binding of transcription factors that interact with chromatin modifiers. PLoS Comput. Biol..

[48] Belch Y, Yang J, Liu Y, Malkaram SA, Liu R, Riethoven JJ, Ladunga I (2010). Weakly positioned nucleosomes enhance the transcriptional competency of chromatin. PLoS One 24.

[49] Lehner B (2010). Conflict between noise and plasticity in yeast. PLoS Genet..

[50] Langmead B, Trapnell C, Pop M, Salzberg SL (2009). Ultrafast and memory-efficient alignment of short DNA sequences to the human genome. Genome Biol..

[51] Gentleman RC, Carey VJ, Bates DM, Bolstad B, Dettling M, Dudoit S, Ellis B, Gautier L, Ge Y, Gentry J (2004). Bioconductor: open software development for computational biology and bioinformatics. Genome Biol..

[52] Flores O, Orozco M (2011). nucleR: a package for non-parametric nucleosome positioning. Bioinformatics.

[53] Olson WK (1998). DNA sequence-dependent deformability deduced from protein-DNA crystal complexes. Proc. Natl Acad. Sci..

[54] Lankas F, Sponer J, Langowski J, Cheatham TE (2003). DNA basepair step deformability inferred from molecular dynamics simulations. Biophys. J..

[55] Lavery R, Zakrzewska K, Beveridge D, Bishop TC, Case DA, Cheatham T, Dixit S, Jayaram B, Lankas F, Laughton C (2010). A systematic molecular dynamics study of nearest-neighbor effects on base pair and base pair step conformations and fluctuations in B-DNA. Nucleic Acids Res..

[56] Pérez A, Lankas F, Luque FJ, Orozco M (2008). Towards a molecular dynamics consensus view of B-DNA flexibility. Nucleic Acids Res..

[57] Pérez A, Marchán I, Svozil D, Sponer J, Cheatham TE, Laughton CA, Orozco M (2007). Refinement of the AMBER force field for nucleic acids: improving the description of alpha/gamma conformers. Biophys. J..

[58] Davey CA, Sargent DF, Luger K, Maeder AW, Richmond TJ (2002). Solvent Mediated Interactions in the Structure of the Nucleosome Core Particle at 1.9Å Resolution†. J. Mol. Biol..

[59] Bryne JC, Valen E, Tang M-HE, Marstrand T, Winther O, da Piedade I, Krogh A, Lenhard B, Sandelin A (2008). JASPAR, the open access database of transcription factor-binding profiles: new content and tools in the 2008 update. Nucleic Acids Res..

[60] Portella G, Battistini F, Orozco M (2013). Understanding the connection between epigenetic DNA methylation and nucleosome positioning from computer simulations. PLoS Comput. Biol..

[61] Gauthier NP, Jensen LJ, Wernersson R, Brunak S, Jensen TS (2010). Cyclebase.org: version 2.0, an updated comprehensive, multi-species repository of cell cycle experiments and derived analysis results. Nucleic Acids Res..

[62] Dabney J, Meyer M (2012). Length and GC-biases during sequencing library amplification: a comparison of various polymerase-buffer systems with ancient and modern DNA sequencing libraries. Biotechniques.

[63] Luger K, Rechsteiner TJ, Flaus AJ, Waye MM, Richmond TJ (1997). Characterization of nucleosome core particles containing histone proteins made in bacteria. J. Mol. Biol..

[64] Luger K (2006). Dynamic nucleosomes. Chromosome Res..

