# Supplementary material for: Fuzziness and noise in nucleosomal architecture
Source: Nucleic Acids Res. 2014 Feb 27;42(8):4934–46. doi: 10.1093/nar/gku165 (PMC4005669; doi:10.1093/nar/gku165)
Supplement: Supplementary Data [file supp_42_8_4934__index.html]

Fuzziness and noise in nucleosomal architecture — Fuzziness and noise in nucleosomal architecture — Supplementary Data 

# Fuzziness and noise in nucleosomal architecture

## Supplementary Data

files

**Files in this Data Supplement:**

- Supplementary Data - pdf file
