# Supplementary material for: Fuzziness and noise in nucleosomal architecture
Source: Nucleic Acids Res. 2014 Feb 27;42(8):4934–46. doi: 10.1093/nar/gku165 (PMC4005669; doi:10.1093/nar/gku165)
Supplement: Supplementary Data [file supp_gku165_nar-00256-x-2014-File007.pdf]

# FUZZINESS AND NOISE IN NUCLEOSOMAL ARCHITECTURE

Oscar Flores, Özgen Deniz, Montserrat Soler-López and Modesto Orozco

## SUPPLEMENTARY TABLES

| default | score_w | 0.6 | | score_h | 0.4 | | |
|---|---|---|---|---|---|---|---|
| | FcF | FoF | WcW | WoW | M-F | M--W | +1_missing |
| R1 (2x) | 226 | 89 | 1808 | 1674 | 106 | 605 | 42 |
| R2 (2x) | 183 | 59 | 1942 | 1606 | 86 | 464 | 20 |
| As (2x) | 277 | 167 | 1624 | 1419 | 171 | 658 | 34 |
| Ov (2x) | 204 | 205 | 1405 | 1285 | 314 | 840 | 56 |
| Un (2x) | 478 | 358 | 763 | 623 | 211 | 361 | 15 |
| **var1** | **score_w** | **0.5** | | **score_h** | **0.5** | | |
| | FcF | FoF | WcW | WoW | M-F | M--W | +1_missing |
| R1 (2x) | 149 | 63 | 1785 | 1618 | 103 | 608 | 42 |
| R2 (2x) | 109 | 46 | 1916 | 1574 | 74 | 476 | 20 |
| As (2x) | 136 | 113 | 1722 | 1476 | 133 | 696 | 34 |
| Ov (2x) | 211 | 210 | 1261 | 1147 | 345 | 809 | 56 |
| Un (2x) | 360 | 273 | 853 | 677 | 174 | 398 | 15 |
| **var2** | **score_w** | **0.4** | | **score_h** | **0.6** | | |
| | FcF | FoF | WcW | WoW | M-F | M--W | +1_missing |
| R1 (2x) | 287 | 152 | 1433 | 1362 | 149 | 562 | 42 |
| R2 (2x) | 187 | 79 | 1643 | 1415 | 104 | 446 | 20 |
| As (2x) | 238 | 185 | 1410 | 1259 | 194 | 635 | 34 |
| Ov (2x) | 341 | 354 | 1016 | 935 | 424 | 730 | 56 |
| Un (2x) | 431 | 335 | 726 | 596 | 198 | 374 | 15 |

**Supplementary Table S1.** Nucleosomes call evaluation based on nucleR TSS clustering by applying different thresholds. The number of genes clustered in the main nucleosome pattern annotations are displayed according to the default parameters score_w = 0.6 and score_h=0.4; score_w=0.5 and score_h=0.5 (*var1*); and score_w=0.4 and score_h=0.6 (*var2*). Key: **R1 (2x)** – replica 1 paired-end dataset; **R2** – replica 2; **As** – asynchronized; **Ov** – MNase-overdigested; **Un** – underdigested.

| | -1 Nucleosome | | | NFR | | +1 Nucleosome | |
|---|---|---|---|---|---|---|---|
| | M | F | W | Closed | Open | F | W |
| R1 (1x) | 648<br>9.68% | 2544<br>38.02% | 2992<br>44.71% | 2858<br>42.71% | 2207<br>32.98% | 2405<br>35.94% | 3779<br>56.47% |
| R2 (1x) | 239<br>3.57% | 2094<br>31.29% | 395<br>59.06% | 3726<br>55.68% | 1860<br>27.79% | 1390<br>20.77% | 4895<br>73.15% |
| R1 (2x) | 711<br>10.62% | 1508<br>22.53% | 4021<br>60.09% | 2939<br>43.92% | 2300<br>34.37% | 1029<br>15.38% | 5211<br>77.87% |
| R2 (2x) | 550<br>8.22% | 1070<br>15.99% | 3852<br>57.56% | 2742<br>40.97% | 2040<br>30.48% | 684<br>10.22% | 4788<br>71.55% |
| As. (2x) | 829<br>12.39% | 1733<br>25.9% | 3656<br>54.63% | 2794<br>41.75% | 2225<br>33.25% | 1302<br>19.46% | 4916<br>73.46% |
| Ov. (2x) | 1154<br>17.24% | 1886<br>28.18% | 3235<br>48.34% | 2559<br>38.24% | 2328<br>34.79% | 1321<br>19.74% | 4954<br>74.03% |
| Un. (2x) | 572<br>8.55% | 2530<br>37.81% | 2051<br>30.65% | 2302<br>34.4% | 1752<br>26.18% | 1846<br>27.59% | 3307<br>49.42% |

**Supplementary Table S2.** Distribution of -1/+1 nucleosomes and NFRs classification for each sample. -1 nucleosomes are classified as missing (M), fuzzy (F) or well-positioned (W) and +1 nucleosomes are either F or W. NFRs can have open or closed configuration depending on the NFR width. Key: **1x** – Single End, **2x** – Paired End, **R1** – Replica 1, **R2** – Replica2, **As** – Asynchronous, **Ov** – Overdigested, **Un** – Underdigested.

| | Same classification | | | | | Variable classification | | | | |
|---|---|---|---|---|---|---|---|---|---|---|
| vs. | Coverage | Cluster | -1 Nuc. | +1 Nuc. | NFR | Coverage | Cluster | -1 Nuc. | +1 Nuc. | NFR |
| R1 (1x)<br>R1 (2x) | 6311<br>(94.31%) | 2552<br>(38.14%) | 4109<br>(61.4%) | 4261<br>(63.67%) | 4599<br>(68.72%) | 23<br>(0.34%) | 1513<br>(22.61%) | 489<br>(7.31%) | 448<br>(6.69%) | 390<br>(5.83%) |
| R2 (1x)<br>R2 (2x) | 5969<br>(89.2%) | 2399<br>(35.85%) | 3480<br>(52%) | 4301<br>(64.27%) | 3751<br>(56.05%) | 63<br>(0.94%) | 1514<br>(22.62%) | 791<br>(11.82%) | 335<br>(5.01%) | 591<br>(8.83%) |
| R1 (1x)<br>R2 (1x) | 5418<br>(80.96%) | 2014<br>(30.1%) | 3606<br>(53.89%) | 4205<br>(62.84%) | 4091<br>(61.13%) | 222<br>(3.32%) | 1994<br>(29.8%) | 1208<br>(18.05%) | 716<br>(10.7%) | 710<br>(10.61%) |
| R1 (2x)<br>R2 (2x) | 5980<br>(89.36%) | 3181<br>(47.53%) | 4068<br>(60.79%) | 4529<br>(67.68%) | 4260<br>(63.66%) | 93<br>(1.39%) | 992<br>(14.82%) | 433<br>(6.47%) | 470<br>(7.02%) | 231<br>(3.45%) |
| R1 (2x)<br>As (2x) | 5123<br>(76.55%) | 2964<br>(44.29%) | 4131<br>(61.73%) | 4758<br>(71.1%) | 4482<br>(66.98%) | 228<br>(3.41%) | 1563<br>(23.36%) | 858<br>(12.82%) | 819<br>(12.24%) | 322<br>(4.81%) |
| R2 (2x)<br>As (2x) | 5781<br>(86.39%) | 2927<br>(43.74%) | 3874<br>(57.89%) | 4393<br>(65.65%) | 4157<br>(62.12%) | 120<br>(1.79%) | 1144<br>(17.1%) | 616<br>(9.21%) | 611<br>(9.13%) | 212<br>(3.17%) |
| R1 (2x)<br>Ov (2x) | 5616<br>(83.92%) | 2881<br>(43.05%) | 3966<br>(59.26%) | 4808<br>(71.85%) | 4452<br>(66.53%) | 91<br>(1.36%) | 1649<br>(24.64%) | 840<br>(12.55%) | 857<br>(12.81%) | 322<br>(4.81%) |
| R2 (2x)<br>Ov (2x) | 5874<br>(87.78%) | 2754<br>(41.15%) | 3612<br>(53.97%) | 4443<br>(66.39%) | 4155<br>(62.09%) | 106<br>(1.58%) | 1181<br>(17.65%) | 680<br>(10.16%) | 670<br>(10.01%) | 217<br>(3.24%) |
| R1 (2x)<br>Un (2x) | 4177<br>(62.42%) | 1377<br>(20.58%) | 2597<br>(38.81%) | 3426<br>(51.2%) | 2917<br>(43.59%) | 405<br>(6.05%) | 2111<br>(31.55%) | 1179<br>(17.62%) | 1090<br>(16.29%) | 705<br>(10.53%) |
| R2 (2x)<br>Un (2x) | 4971<br>(74.28%) | 1355<br>(20.25%) | 2376<br>(35.51%) | 3202<br>(47.85%) | 2767<br>(41.35%) | 221<br>(3.3%) | 1820<br>(27.2%) | 1184<br>(17.69%) | 1047<br>(15.65%) | 586<br>(8.76%) |
| Ov (2x)<br>Un (2x) | 6342<br>(94.77%) | 1915<br>(28.62%) | 3105<br>(46.4%) | 3737<br>(55.84%) | 3279<br>(49%) | 19<br>(0.28%) | 1661<br>(24.82%) | 721<br>(10.77%) | 731<br>(10.92%) | 547<br>(8.17%) |
| As (2x)<br>Ov (2x) | 6421<br>(95.95%) | 3315<br>(49.54%) | 4381<br>(65.47%) | 5033<br>(75.21%) | 4776<br>(71.37%) | 14<br>(0.21%) | 1229<br>(18.37%) | 414<br>(6.19%) | 484<br>(7.23%) | 259<br>(3.87%) |
| As (2x)<br>Un (2x) | 5890<br>(88.02%) | 1703<br>(25.45%) | 2876<br>(42.98%) | 3648<br>(54.51%) | 3065<br>(45.8%) | 79<br>(1.18%) | 1908<br>(28.51%) | 982<br>(14.67%) | 882<br>(13.18%) | 620<br>(9.26%) |

**Supplementary Table S3.** Different pair-wise metrics of nucleosome similarity/dissimilarity. In order to obtain robust estimations of the similarity/dissimilarity of gene architectures between samples we defined the following metrics. *Coverage*: A gene is considered as stable if Pearson's correlation between two samples in the window -300:300 from the TSS is greater than 0.7; is considered as variable if correlation is smaller than 0.5. *Cluster*: We consider a gene stable if the cluster stays the same; we considered a significant variable architecture when 2 of the clustering dimensions (-1/NFR/+1) vary between samples. *+1/-1 Nucleosome*: We consider a nucleosome in the same classification if nucleR's classification is the same for two samples; we considered a gene variable if the absolute difference in nucleR's score is bigger than 0.25 points. *NFR*: we consider a gene stable if the classification of the NFR is the same (open/close/overlap/missing); we considered a change as significant if the change in distance between -1/+1 nucleosomes is more than 100bp. Genes which do not satisfy any of the two criteria are in considered out of the stability/variability threshold. Percentages are relative to the total number of genes in the SacCer3 genome. Key: **1x** – Single End, **2x** – Paired End, **R1** – Replica 1, **R2** – Replica2, **As** – Asynchronous, **Ov** – Overdigested, **Un** – Underdigested.

| | Stable classification | | | Variable classification | | |
|---|---|---|---|---|---|---|
| vs. | Coverage | ∆calls | ∆score | Coverage | ∆calls | ∆score |
| R1 (1x)<br>R1 (2x) | 5688<br>(85%) | 3077<br>(45.98%) | 6010<br>(89.81%) | 169<br>(2.53%) | 446<br>(6.66%) | 33<br>(0.49%) |
| R2 (1x)<br>R2 (2x) | 5436<br>(81.23%) | 3142<br>(46.95%) | 5862<br>(87.6%) | 194<br>(2.9%) | 679<br>(10.15%) | 39<br>(0.58%) |
| R1 (1x)<br>R2 (1x) | 3776<br>(56.43%) | 2976<br>(44.47%) | 5634<br>(84.19%) | 486<br>(7.26%) | 386<br>(5.77%) | 59<br>(0.88%) |
| R1 (2x)<br>R2 (2x) | 5212<br>(77.88%) | 3261<br>(48.73%) | 5759<br>(86.06%) | 341<br>(5.1%) | 577<br>(8.62%) | 53<br>(0.79%) |
| R1 (2x)<br>As (2x) | 3190<br>(47.67%) | 2901<br>(43.35%) | 4958<br>(74.09%) | 869<br>(12.99%) | 461<br>(6.89%) | 96<br>(1.43%) |
| R2 (2x)<br>As (2x) | 4578<br>(68.41%) | 2971<br>(44.4%) | 5461<br>(81.6%) | 459<br>(6.86%) | 537<br>(8.02%) | 72<br>(1.08%) |
| R1 (2x)<br>Ov (2x) | 4297<br>(64.21%) | 3096<br>(46.26%) | 5129<br>(76.64%) | 463<br>(6.92%) | 417<br>(6.23%) | 114<br>(1.7%) |
| R2 (2x)<br>Ov (2x) | 5047<br>(75.42%) | 3448<br>(51.52%) | 5462<br>(81.62%) | 427<br>(6.38%) | 321<br>(4.8%) | 91<br>(1.36%) |
| R1 (2x)<br>Un (2x) | 2550<br>(38.11%) | 2493<br>(37.25%) | 3938<br>(58.85%) | 1176<br>(17.57%) | 769<br>(11.49%) | 262<br>(3.92%) |
| R2 (2x)<br>Un (2x) | 3678<br>(54.96%) | 2647<br>(39.55%) | 4279<br>(63.94%) | 767<br>(11.46%) | 508<br>(7.59%) | 224<br>(3.35%) |
| Ov (2x)<br>Un (2x) | 6058<br>(90.53%) | 2786<br>(41.63%) | 5631<br>(84.15%) | 121<br>(1.81%) | 459<br>(6.86%) | 48<br>(0.72%) |
| As (2x)<br>Ov (2x) | 6101<br>(91.17%) | 3014<br>(45.04%) | 6094<br>(91.06%) | 91<br>(1.36%) | 554<br>(8.28%) | 17<br>(0.25%) |
| As (2x)<br>Un (2x) | 5280<br>(78.9%) | 2462<br>(36.79%) | 5294<br>(79.11%) | 237<br>(3.54%) | 828<br>(12.37%) | 101<br>(1.51%) |

**Supplementary Table S4.** Different pair-wise metrics of nucleosome similarity/dissimilarity in gene body regions. *Coverage*: a particular gene is considered 'stable' when Pearson's correlation between the whole gene body of two samples is greater than 0.7; otherwise, it is considered 'variable' when correlation is smaller than 0.5. Δ*calls*: a gene is considered 'stable' when it has the same number of nucleosome calls inside the gene body; otherwise, it is considered 'variable' when the difference of nucleosome calls between samples is more than 2 nucleosomes. Δ*score*: a particular gene is considered to be the same between two samples when the equivalent nucleosome calls have a mean difference of nucleR score lower than 0.15; otherwise, the gene is assigned as variable when this difference is higher than 0.25. Genes which do not satisfy any of the two criteria are in considered out of the stability/variability threshold. Percentages are relative to the total number of genes in the SacCer3 genome. *Note: the present metrics differ from those presented in the previous Supplementary Table 3, in order to account for an arbitrary number of nucleosome calls in gene body regions.* Key: **1x** – Single End, **2x** – Paired End, **R1** – Replica 1, **R2** – Replica2, **As** – Asynchronous, **Ov** – Overdigested, **Un** – Underdigested.

| | Stable classification | | | Variable classification | | |
|---|---|---|---|---|---|---|
| **vs.** | **Coverage** | **∆calls** | **∆score** | **Coverage** | **∆calls** | **∆score** |
| **R1 (1x) R1 (2x)** | 6210 (92.80%) | 3965 (59.25%) | 5868 (87.69%) | 81 (1.21%) | 227 (3.39%) | 27 (0.4%) |
| **R2 (1x) R2 (2x)** | 5944 (88.82%) | 2893 (43.23%) | 5729 (85.61%) | 118 (1.76%) | 830 (12.4%) | 50 (0.75%) |
| **R1 (1x) R2 (1x)** | 5455 (81.52%) | 3411 (50.97%) | 5220 (78%) | 273 (4.08%) | 82 (1.23%) | 92 (1.37%) |
| **R1 (2x) R2 (2x)** | 5929 (88.60%) | 4397 (65.71%) | 5614 (83.89%) | 174 (2.6%) | 281 (4.2%) | 76 (1.14%) |
| **R1 (2x) As (2x)** | 5136 (76.75%) | 3907 (58.38%) | 4825 (72.10%) | 364 (5.44%) | 54 (0.81%) | 176 (2.63%) |
| **R2 (2x) As (2x)** | 5738 (85.74%) | 4303 (64.30%) | 5366 (80.19%) | 242 (3.62%) | 92 (1.37%) | 108 (1.61%) |
| **R1 (2x) Ov (2x)** | 5562 (83.11%) | 3885 (58.05%) | 4787 (71.53%) | 209 (3.12%) | 252 (3.77%) | 179 (2.67%) |
| **R2 (2x) Ov (2x)** | 5820 (86.97%) | 4477 (66.90%) | 5109 (76.34%) | 223 (3.33%) | 87 (1.3%) | 118 (1.76%) |
| **R1 (2x) Un (2x)** | 4344 (64.91%) | 2890 (43.19%) | 3928 (58.70%) | 649 (9.7%) | 179 (2.67%) | 447 (6.68%) |
| **R2 (2x) Un (2x)** | 4975 (74.34%) | 3000 (44.83%) | 3880 (57.98%) | 445 (6.65%) | 160 (2.39%) | 453 (6.77%) |
| **Ov (2x) Un (2x)** | 6248 (93.37%) | 3248 (48.54%) | 5254 (78.51%) | 67 (1%) | 118 (1.76%) | 87 (1.3%) |
| **As (2x) Ov (2x)** | 6353 (94.93%) | 4059 (60.65%) | 5982 (89.39%) | 43 (0.64%) | 290 (4.33%) | 33 (0.49%) |
| **As (2x) Un (2x)** | 5837 (87.22%) | 3009 (44.96%) | 4858 (72.59%) | 163 (2.44%) | 149 (2.23%) | 170 (2.54%) |

**Supplementary Table S5**. Different pair-wise metrics of nucleosome similarity/dissimilarity around TSSs (window -300:+300). Metrics used here are the same than in Supplementary Table S4.

# SUPPLEMENTARY FIGURES

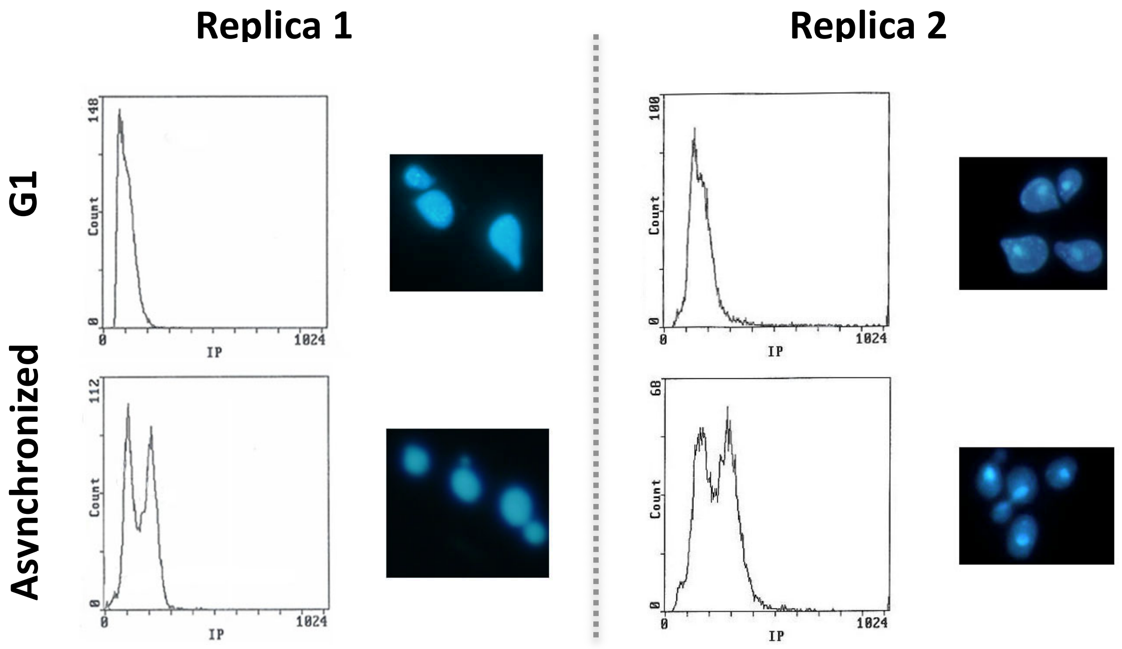


**Supplementary Figure S1.** Flow cytometry analysis and fluorescence microscope images of late G1 synchronized cells (upper panel) and asynchronous cells (lower panel) for *replicas* 1 (left) and 2 (right).

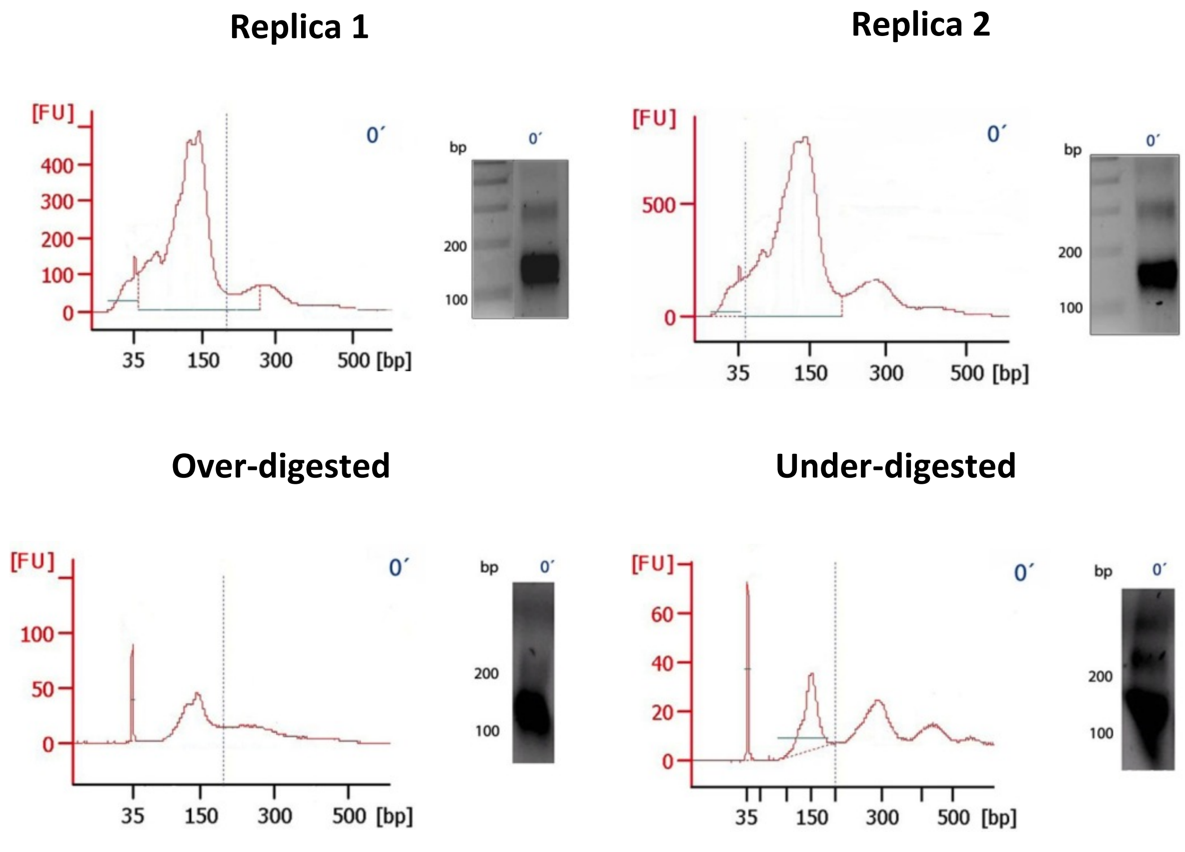


**Supplementary Figure S2.** MNase digestion profiles  of replica 1 (top-left), replica 2 (top-rigth), over-digested sample (bottom-left) and under-digested sample (bottom-right). The left panels show the size distribution of digested DNA molecules as measured by Bioanalyzer and the right panels show the agarose gel analysis of digestion products.

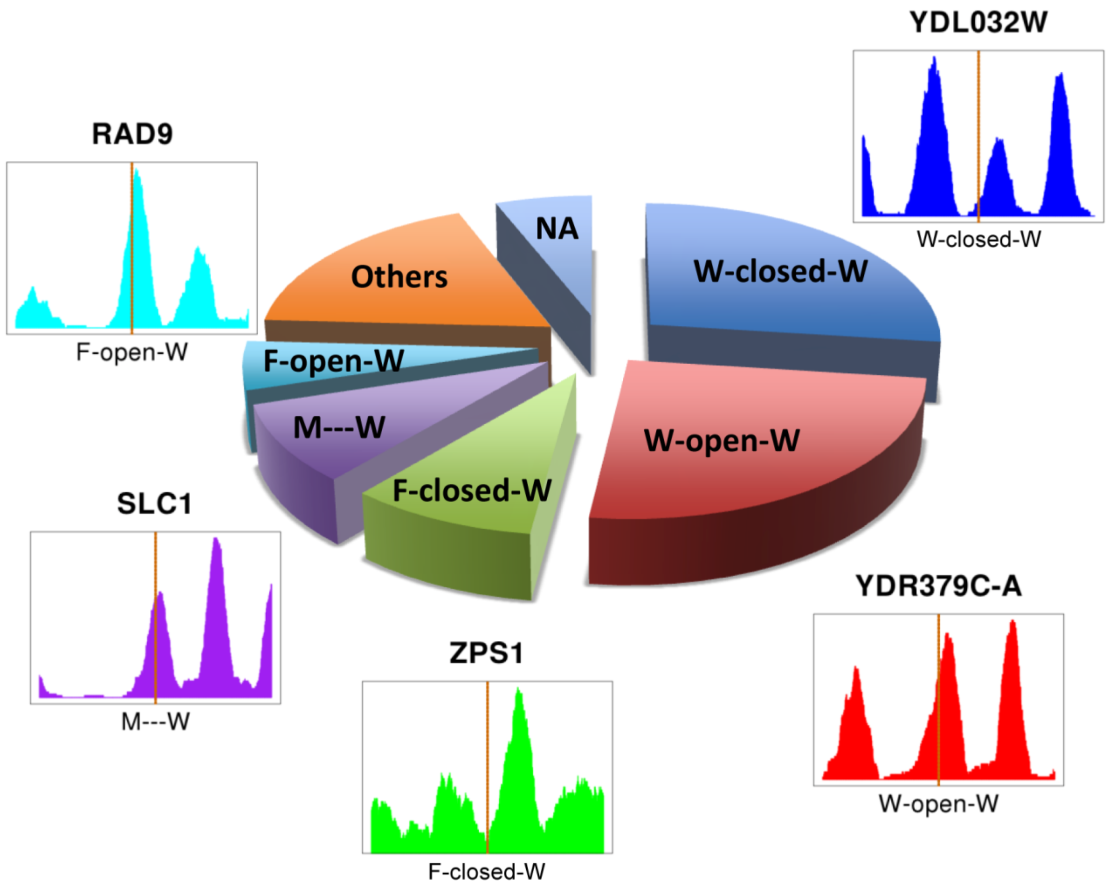


**Supplementary Figure S3. Gene clustering according to nucleosomal architecture at transcription start sites.** Pie-chart shows the gene distribution for the most populated classes in the sample Replica 2 (2x). For every class, an example of the nucleosome coverage around the TSS of a representative gene is illustrated (window -300:300 from the TSS, marked in red). All plots show the coverage in 5'->3' direction, representing the +1 nucleosome as the peak overlapping or immediately downstream TSS and the -1 nucleosome as the peak right upstream of +1. In the case of SLC1, -1 nucleosome peak is not detected in the -300:300 window.

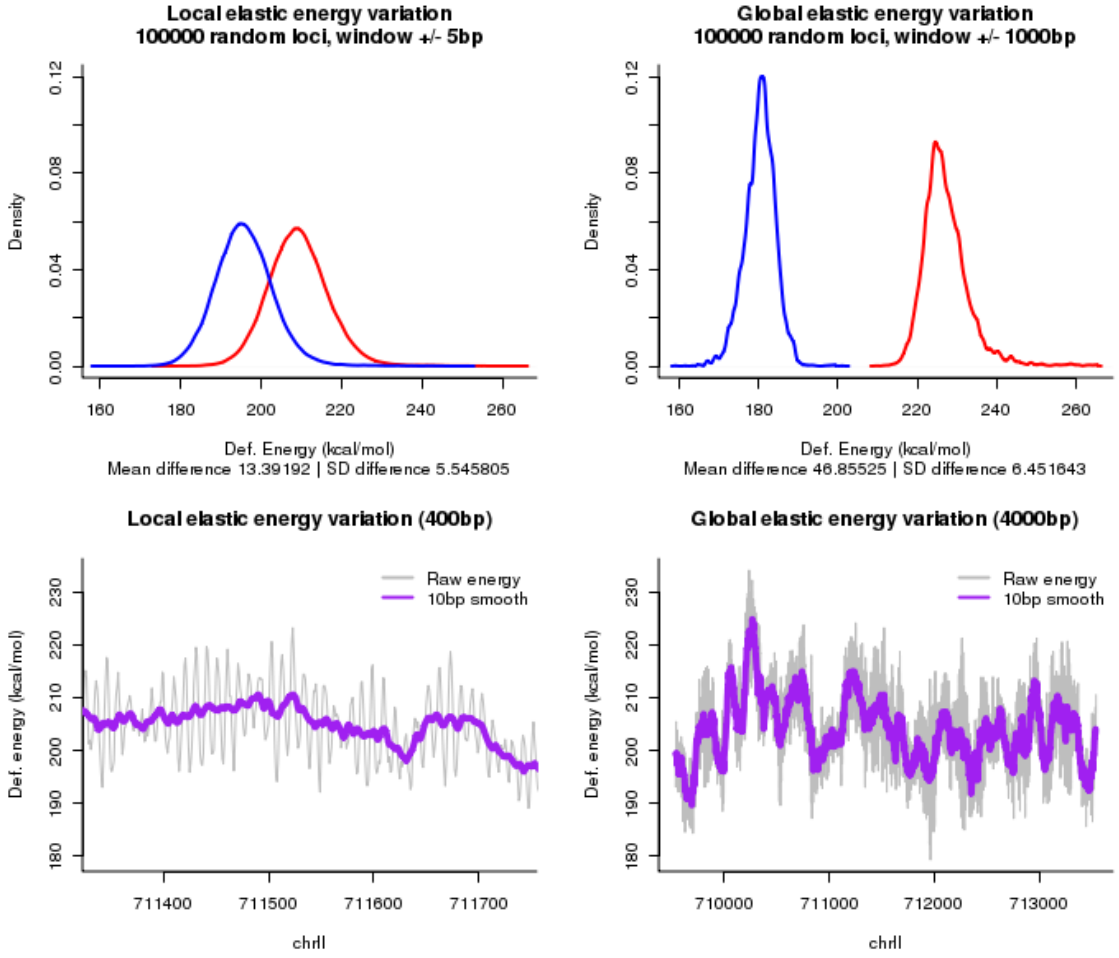


**Supplementary Figure S4. Local and global energy variation.** Despite local energy variation involves a strong periodicity of 10bp with small energy fluctuations (around 13.39 kcal/mol) (left), these don't act as a strong regulator of the nucleosome fuzziness. Global energy barriers with a larger mesoscopic effect (around 46.86 kcal/mol) could act as intrinsic regulator of the nucleosome phasing along different cells. On top, minimum (blue) and maximum (red) values in a window of +/- 5bp (left) and +/- 1000bp right of 100000 random loci. On the bottom, we show the raw energy (grey) and the 10bp average (purple) of single random region of the chromosome II (left: 400bp window, right:4000bp window).

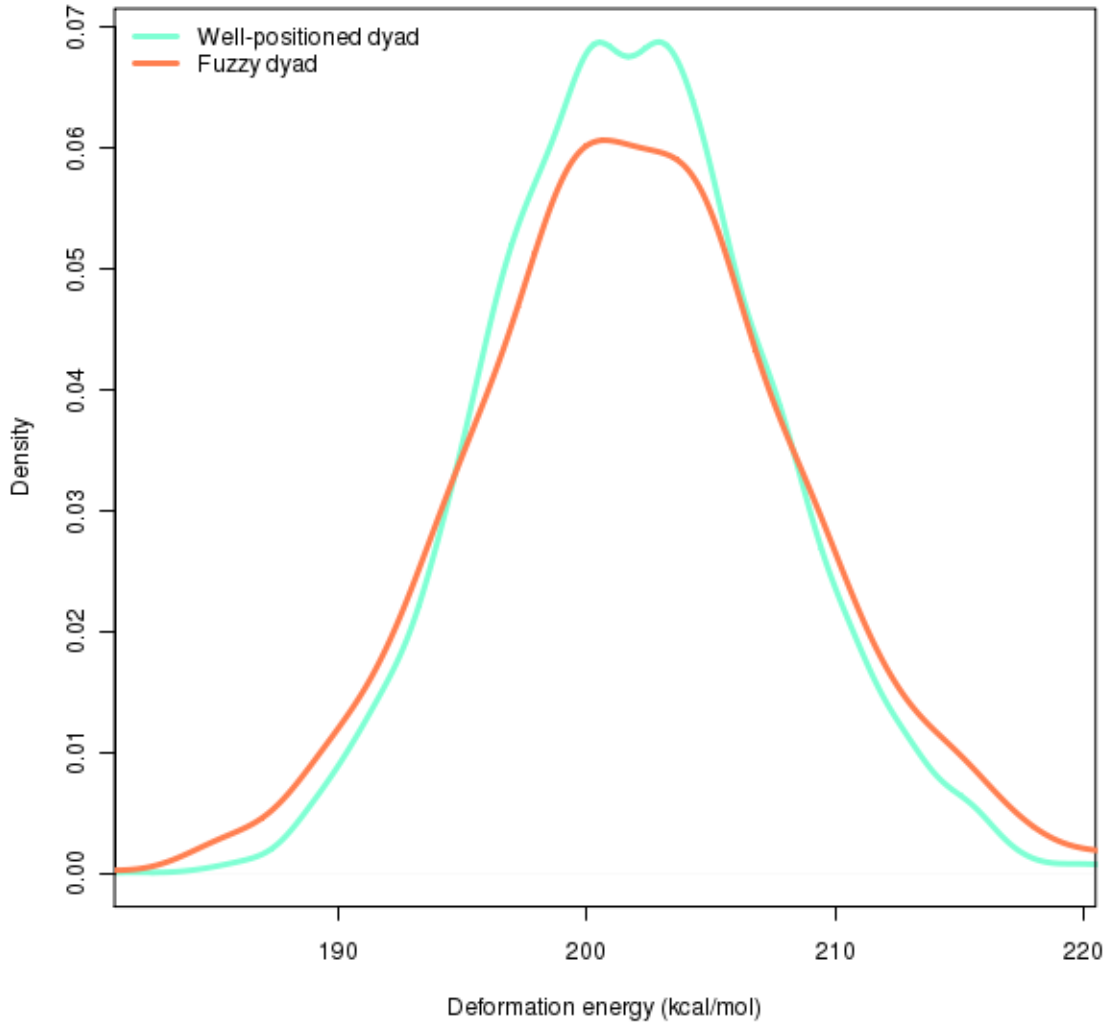


**Supplementary Figure S5. Deformation energy of well-positioned and fuzzy nucleosomes.** Deformation energy around +/-5bp around the peak summit has been calculated for annotated -1/+1 nucleosomes. Mean value of every 10 possible combinations was used to account for local periodicity. Fuzzy and well-positioned nucleosomes are taken from the common ones in *replicas* 1 and 2.

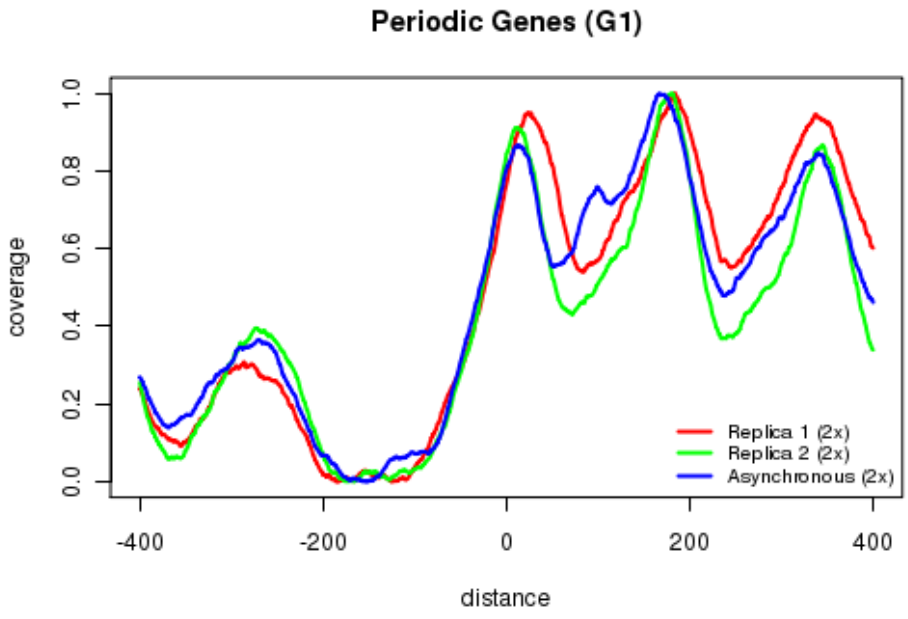


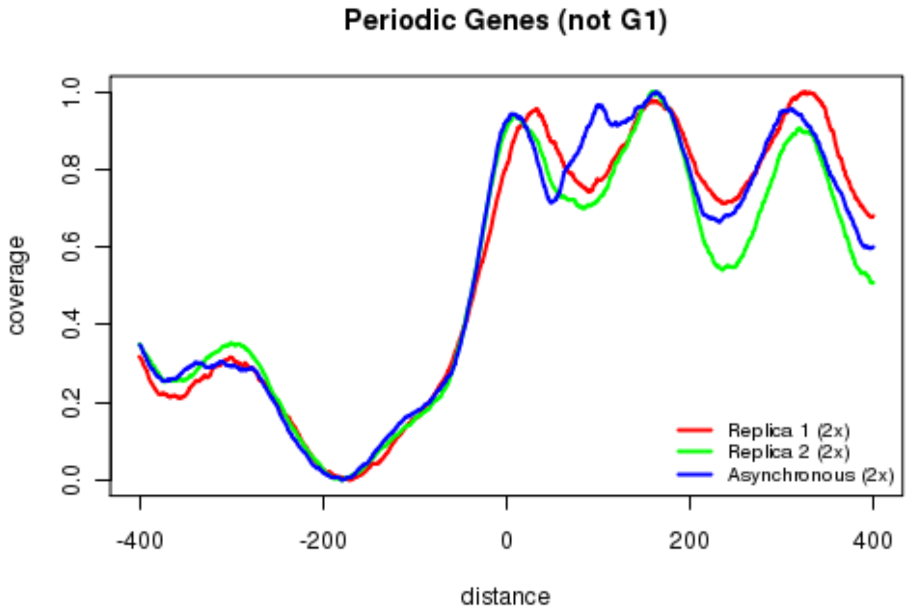


**Supplementary Figure S6. Effect of cell-cycle periodic genes in nucleosome map.** Coverage of cell-cycle periodic genes is shown for G1 related genes (top, 211 genes) and in other stages (bottom, 365 genes). Asynchronous sample (blue) shows a larger perturbation between the +1/-1 nucleosome peaks in both cases.

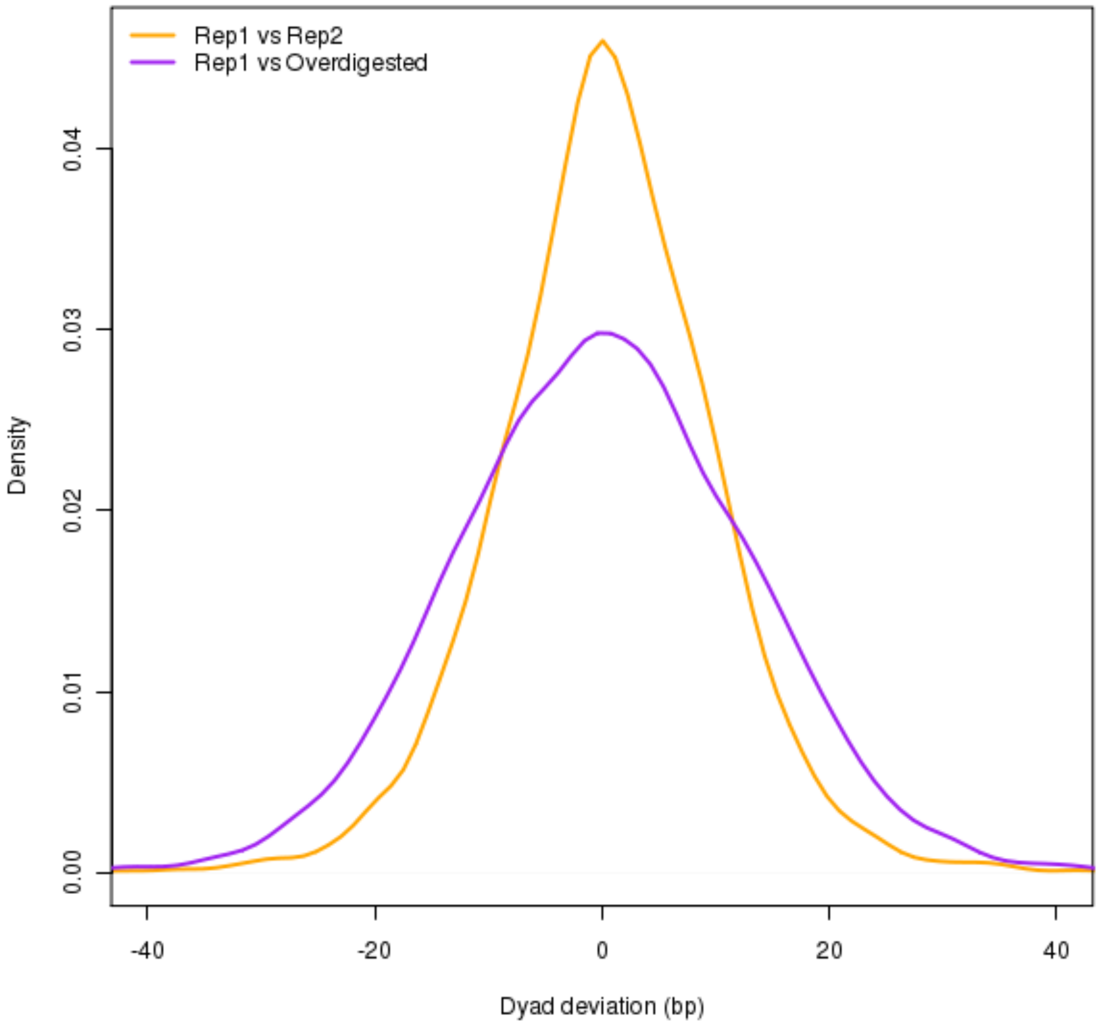


**Supplementary Figure S7. Comparison of dyad deviations due to different digestion.** Dyad distances of annotated -1/+1 nucleosomes (coverage peak summits) have been calculated between biological replicates and over-digested sample. Absolute mean deviation between Rep1 and Rep2 is 14.25 bp, compared with 18.75bp (+4.5bp) in the case of Rep1 with over-digested sample.
